# The mitochondrial inhibitor IF1 binds to the ATP synthase OSCP subunit and protects cancer cells from apoptosis

**DOI:** 10.1038/s41419-023-05572-y

**Published:** 2023-01-23

**Authors:** Chiara Galber, Simone Fabbian, Cristina Gatto, Martina Grandi, Stefania Carissimi, Manuel Jesus Acosta, Gianluca Sgarbi, Natascia Tiso, Francesco Argenton, Giancarlo Solaini, Alessandra Baracca, Massimo Bellanda, Valentina Giorgio

**Affiliations:** 1 Department of Biomedical and Neuromotor Sciences, University of Bologna, Bologna, I-40126 Italy; 2 Consiglio Nazionale delle Ricerche Institute of Neuroscience, Padova, I-35121 Italy; 3 Department of Chemical Science, University of Padova, Padova, I-35121 Italy; 4 Department of Biology, University of Padova, Padova, I-35131 Italy; 5 Consiglio Nazionale delle Ricerche Institute of Biomolecular Chemistry, Padova, I-35131 Italy

**Keywords:** Apoptosis, Structural biology

## Abstract

The mitochondrial protein IF1 binds to the catalytic domain of the ATP synthase and inhibits ATP hydrolysis in ischemic tissues. Moreover, IF1 is overexpressed in many tumors and has been shown to act as a pro-oncogenic protein, although its mechanism of action is still debated. Here, we show that *ATP5IF1* gene disruption in HeLa cells decreases colony formation in soft agar and tumor mass development in xenografts, underlining the role of IF1 in cancer. Notably, the lack of IF1 does not affect proliferation or oligomycin-sensitive mitochondrial respiration, but it sensitizes the cells to the opening of the permeability transition pore (PTP). Immunoprecipitation and proximity ligation analysis show that IF1 binds to the ATP synthase OSCP subunit in HeLa cells under oxidative phosphorylation conditions. The IF1–OSCP interaction is confirmed by NMR spectroscopy analysis of the recombinant soluble proteins. Overall, our results suggest that the IF1-OSCP interaction protects cancer cells from PTP-dependent apoptosis under normoxic conditions.

## Introduction

Mitochondria contain a 10 kDa heat-stable protein, inhibitor factor 1 (IF1), which is a reversible non-competitive inhibitor of ATP hydrolysis [1]. The role of IF1 consists in limiting ATP dissipation, under the condition of oxygen deprivation, as seen in heart ischemia [2–4]. Two regions of the mature IF1 protein have been the focus of structure-function studies. The first is the N-terminal region which inhibits a single ATPase at a catalytic interface between the βDP and αDP subunits [5–9]. The second is the C-terminal region, which stabilizes IF1 homo-dimers forming an anti-parallel coiled coil [10]. The interaction of IF1 dimers with dimeric ATP synthase complexes has been resolved in a recent cryo-EM structure [11]. Self-association of IF1 dimers in tetramers involves the N-terminus of the protein hence masking its inhibitory region [12]. The equilibrium between dimers and inactive tetramers of IF1 is crucial in the in vivo modulation of the inhibitory activity and is sensitive to IF1 concentration, pH and ions fluctuations [10].

IF1 may also play a role in promoting cancer development and growth [13, 14]. Its high expression level has been found in different human tumors [15], although the mechanism through which IF1 promotes cancer growth and progression remains debated [16]. Recent findings suggest that IF1 might bind to ATP synthase when the enzyme works physiologically [i.e., ATP synthesis [17–20]] and that its role in preventing ATP hydrolysis is observed only under anoxia/near-anoxia [21]. The possibility of a different mechanism of IF1 binding during ATP synthesis remains to be studied, since the interaction of IF1 with the ATP synthase catalytic subunits requires the hydrolysis of ATP molecules [7, 8]. An alternative IF1 binding site was suggested on the ATP synthase oligomycin sensitivity conferring protein (OSCP) in isolated bovine mitochondria [22]. This hypothesis was never investigated in cancer, however in hepatocellular carcinoma IF1 was shown to promote cell proliferation and colony formation in vitro, by increasing STAT3 level and decreasing the expression of E-cadherin [23]. Another mechanism concerns the effect of IF1 overexpression in keeping stable ATP synthase dimers and high cristae density [24, 25]. Accordingly, a high IF1 level in cancer might counteract the apoptotic process by OPA1 oligomer stabilization, impeding cristae remodeling during apoptosis [26].

Although an anti-apoptotic role has been attributed to IF1, its effect on mitochondrial permeability transition (PT) pore (PTP) modulation has not been investigated. This is a Ca^2+^-dependent high-conductance channel on the inner mitochondrial membrane [27, 28] leading to apoptosis. Many cancer models inhibit cell death in response to fluctuations of the PTP effectors, such as Ca^2+^or Mg^2+^, reactive oxygen species (ROS), and matrix pH [29], or controlling the PTP association with its physiological inducer, the cyclophilin D (CyPD) [30–32], which modulates ATP synthase activity [33], binding its OSCP subunit [30].

ATP synthase complexes have been proposed to contribute to PTP formation [30, 34–39], although their involvement is still debated [40, 41]. Genetic manipulations of enzyme components have revealed modulatory mechanisms of the channel [42–48], but whether the IF1 binding to ATP synthase might have a role in PTP modulation in cancer has not been established so far. Here, we show that IF1 can interact with the ATP synthase OSCP subunit in HeLa cancer cells under normal culture conditions. Moreover, the effect of the IF1-OSCP interaction on PTP-dependent apoptosis was investigated to elucidate its possible role in cancer cell survival.

## Results

### IF1 binds to ATP synthase of cancer cell mitochondria under State 3 respiratory condition

**Fig. 1:**
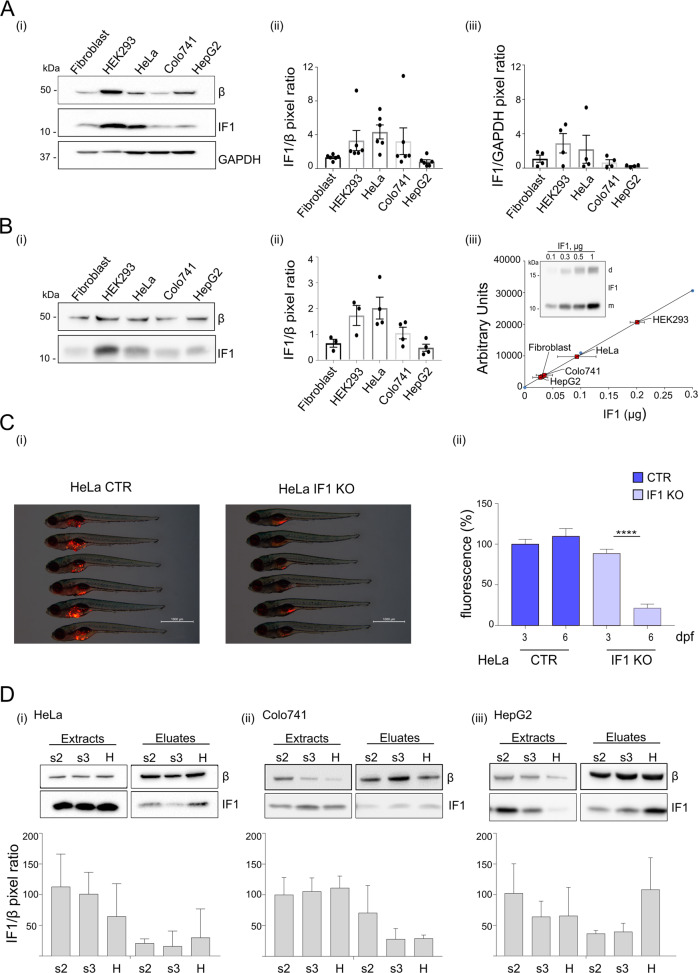
The inhibitor IF1 binds to ATP synthase in cell mitochondria under ATP hydrolysis, State 2 and State 3 steady state respiratory conditions. **A** Western blotting is shown of (i) β subunit IF1 and GAPDH from Fibroblast, HEK293, HeLa, Colo741 and HepG2 cell lysates. Molecular markers are on the left. Histograms of (ii) mean IF1/β subunit or (iii) mean IF1/GAPDH pixel ratio are shown (data are mean ± SEM from six or four independent experiments, respectively). **B** Western blotting is shown of (i) β subunit and IF1 from Fibroblast, HEK293, HeLa, Colo741 and HepG2 mitochondria. In (ii), the histogram of mean IF1/β subunit pixel ratio is shown. In (iii), the quantification is shown of the amount of IF1 in 10 µg of each human cell mitochondrial preparation, based on IF1 bands measured in arbitrary units by densitometry and comparison with the titration of IF1, purified from bovine mitochondria (insert, d is dimeric, m is monomeric IF1). Molecular markers are on the left. Data are mean ± SEM from at least three independent experiments. **C** CTR and IF1 KO HeLa cells, upon fluorescent staining with Vibrant DiI, were injected into the yolk of zebrafish (about 100 cells per embryo) aged 2 days post fertilization (dpf). In (i), images of fish at 6 dpf are shown. Red fluorescence indicates HeLa cell-derived tumor masses. Scale bar, 1000 μm. In (ii), mean HeLa cell fluorescence (%) is shown at 3 (initial fluorescence upon cell injection) and 6 dpf (fluorescence indicates tumor mass development). Data are mean ± SEM from six independent experiments, ****p = 0.000003. **D** Mitochondria isolated from HeLa (i), Colo741 (ii) and HepG2 (iii) cells, are incubated in a buffer promoting state 2 (s2) or state 3 (s3) respiration or ATP hydrolysis (H) and solubilized in a 1 % (w/v) digitonin containing buffer. Mitochondrial extracts were subjected to immunoprecipitation of ATP synthase. Western blotting is shown of β subunit and IF1 protein contained in the digitonin mitochondrial extracts (extracts), and in the immunoprecipitated fractions (eluates). Histograms refer to IF1/β pixel ratio normalized to each s2 extract (set as 100%), bottom panels. Data are mean ± SEM of at least three independent experiments.

Cell lysates and mitochondria from human cancer lines derived from cervix (HeLa), colon (Colo741) adenocarcinomas, liver (HepG2) carcinoma and from human skin fibroblasts and human embryonic kidney (HEK293) cells were studied to evaluate the content of both the inhibitor IF1 and the β subunit of ATP synthase, Fig. 1A, B. The IF1/β ratio of cancer lysates and mitochondria was higher in HeLa than in Colo741 and HepG2 cells, Fig. 1A, B (ii). The active IF1 was quantified in cell mitochondria using a titration curve of purified bovine IF1 as a reference, Fig. 1B (iii). Overall the absolute quantification in Fig. 1B (iii) shows that IF1 mitochondrial content in cancer cell lines is not representative of its increased levels shown in cancer tissues compared to controls [15]. However, HeLa (CTR, expressing IF1) cell injection in zebrafish embryos at 2 days post-fertilization (dpf) caused a tumor mass development in the following days (6 dpf), which was not observed in the IF1 knock out (KO) HeLa-injected embryos, Fig. 1C, confirming an important role of IF1 expression in cancer development in vivo.

Of note, the IF1 KO cells, as well as the IF1 knock down (KD) cells, did not show any difference in the amount of ATP synthase subunits (i), nor in the tested mitochondrial membrane or matrix proteins (ii), compared to controls (Fig. S1A and S1B). IF1 KO and IF1 KD mitochondrial proteins normalized to the cytosolic Glyceraldehyde 3-phosphate dehydrogenase (GAPDH) did not differ from those in their respective controls (Fig. S1A (iii), S1B (iii)).

HeLa, Colo741 and HepG2 cancer lines were further studied here to test whether different physiological conditions (i.e., State 2 respiration, ATP synthesis or ATP hydrolysis) might promote the IF1 binding to ATP synthase.

ATP synthase was immunoprecipitated from mitochondria that were maintained under different functional conditions (stimulated ATP hydrolysis, State 2 and State 3 steady state respiratory conditions). Mitochondrial extracts were obtained applying 1% (w/v) digitonin, resulting in a mixed population of oligomeric, dimeric, and monomeric ATP synthase complexes [45, 49]. IF1 was detected in mitochondrial extracts (before immunoprecipitation) and in anti-ATP synthase immunoprecipitated fractions (eluates, Fig. 1D). These fractions contained IF1, not only when they derived from mitochondria maintained under continuous ATP hydrolysis (H), but also in State 2 (s2) and State 3 (s3) respiration (Fig. 1D). Each mitochondrial preparation was tested before immunoprecipitation and showed that mitochondria were depleted of endogenous substrates (Fig. S1C (i), before succinate) and that they accumulated comparable membrane potential in the three functional conditions above, as previously published [30]. Notably, mitochondrial respiration was stimulated by ADP (compare the slope in State 3 and State 2, Fig. S1C (i)). Since ATP hydrolysis is necessary to allow the IF1 inhibitory binding to the catalytic F1 domain [5, 7], the finding that IF1 binds ATP synthase during State 2 and State 3 respiration suggested the existence of an additional binding site.

### IF1 binds to the OSCP subunit

**Fig. 2:**
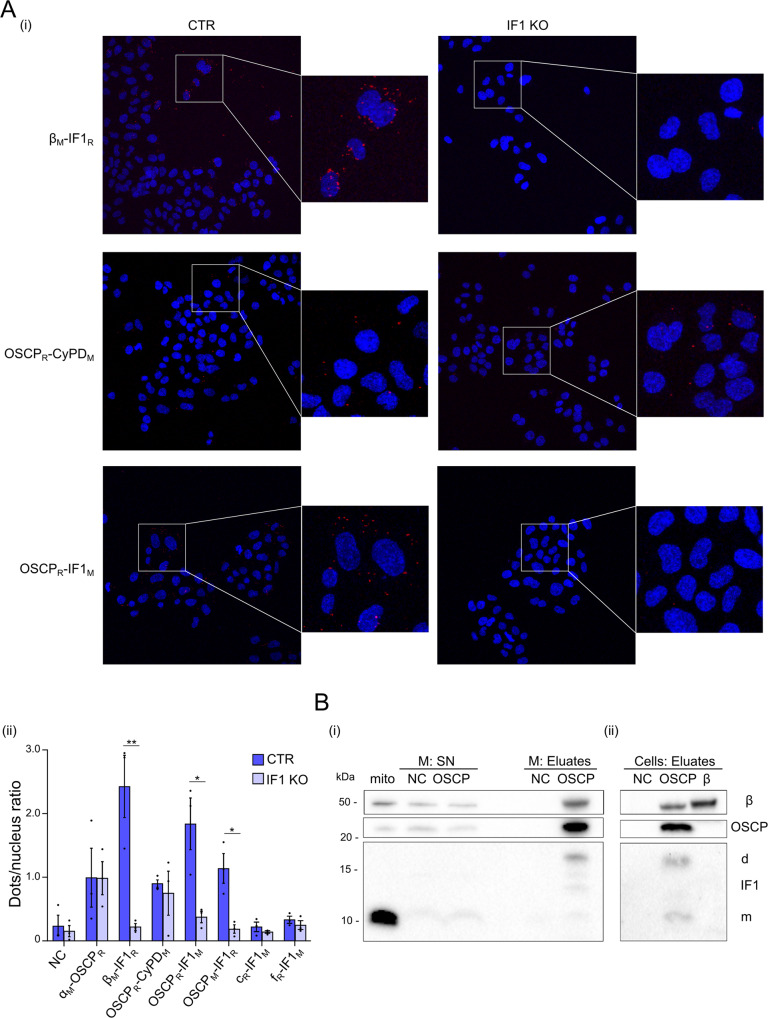
IF1 binds to the OSCP subunit of ATP synthase in situ and under oxidative phosphorylation. **A** Representative images, and their magnification (small panels), of control (CTR) and IF1 knock out (IF1 KO) HeLa cells are analyzed by Proximity Ligation Assay (PLA) to assess protein-protein interactions (i). The antibody combinations applied to test the interactions between mitochondrial proteins are indicated on the left. They detect IF1, β or OSCP subunits and cyclophilin D (CyPD). M or R indicate the secondary antibodies used during the PLA protocol. Interactions are revealed by red dots, while DAPI-stained nuclei are in blue. Images are acquired with a Leica TCS SP5 confocal microscope equipped with a CCD camera and a 40x objective. PLA analysis quantification (ii) is shown of the number of red fluorescent dots per nucleus in CTR and IF1 KO HeLa cells. Data are mean ± SEM of three independent experiments, *p < 0.05, **p = 0.01. **B** Isolated HeLa mitochondria (M, (i)), or permeabilized HeLa cells (Cells, (ii)) are incubated in a buffer promoting state 3 respiration and processed for immunoprecipitation of OSCP (OSCP), or β (β) subunits (NC is negative control). Western blotting is shown recognizing β, OSCP subunits and IF1 (indicated on the right; d is dimeric, m is monomeric IF1), representative of three independent experiments. Molecular markers are shown on the left. Flow through fractions (supernatants, SN) of cell immunoprecipitation are shown in the original blot file.

To investigate the binding site of IF1 on ATP synthase in respiring cancer cells, the possible interactions of IF1 were studied in HeLa cells in situ through the Proximity Ligation Assay (PLA), Fig. 2A and S1D. IF1 KO HeLa cells (Fig. S1A) were used as a negative control for IF1 interactions. The interactions of β subunit-IF1, OSCP-CyPD, OSCP-IF1, and α subunit-OSCP were detected by PLA in control HeLa cells in situ (Fig. 2A, S1D). In IF1 KO cells only the OSCP-CyPD and α subunit–OSCP interactions were found, Fig. 2A (ii), in line with the unaltered amount of CyPD, OSCP and α subunits in these cells (Fig. S1A), and no interaction was detected in the absence of antibodies (Fig. 2A (ii), NC). Normalization of protein interactions (red dots) to the number of nuclei (blue fluorescence, in Fig. 2A and S1D) showed that IF1 interacts with both β and OSCP subunits, but not with the c or f subunits, in control HeLa cells in situ. To further investigate the IF1 interaction with OSCP subunit, freshly prepared HeLa mitochondria were incubated in an ADP-regenerating buffer (State 3 steady state respiratory condition, Fig. 2B (i)), and their extracts were immunoprecipitated for the OSCP subunit. IF1 immunoprecipitated with OSCP when the OSCP antibody was used (OSCP, Fig. 2B (i)), while IF1 was not detected in eluates in the negative control (NC, Fig. 2B (i)). An intense band corresponding to IF1 dimer was detected in the OSCP immunoprecipitated fraction (Fig. 2B (i)), suggesting that the IF1 dimeric or oligomeric form might be involved in this interaction. To further validate the IF1-OSCP interaction during oxidative phosphorylation, permeabilized respiring HeLa cells (State 3) were subjected to immunoprecipitation for the OSCP or the β subunit (Fig. 2B (ii)). The inhibitor IF1 was immunoprecipitated with the OSCP, but did not bind to the β subunit. These findings strongly indicated that the OSCP subunit represents a binding site for IF1 during oxidative phosphorylation.

### The N-terminus of IF1 interacts with the N-terminus of OSCP

**Fig. 3:**
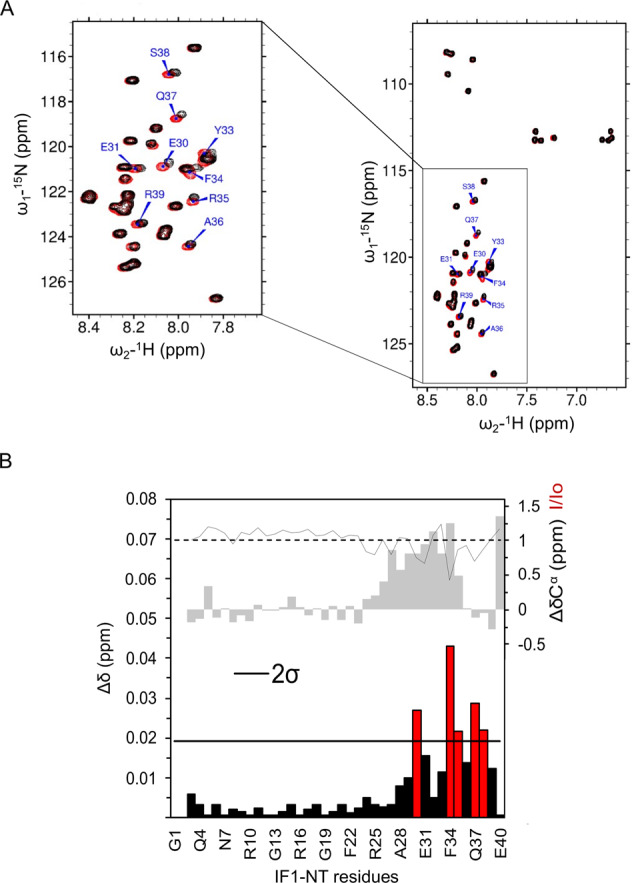
IF1-NT interacts in vitro with OSCP-NT through its N-terminal region. **A** ^1^H-^15^N SOFAST-HMQC spectrum of IF1-NT (residues G1-E40) in the absence (red) and in the presence of a 15-fold molar excess of unlabeled OSCP-NT (residues R6-G114) (black). The expanded plot shows the region of the spectrum containing the cross peaks with pronounced chemical shift perturbation, which are labeled. **B** Combined chemical shift perturbations (colored bars, left axis, bottom panel) derived from the experiments shown in **A**. Red bars indicate residues showing a deviation larger than 2 standard deviations (2*σ*) from the average. The gray line on the top of the panel represents the normalized signal intensity changes in IF1-NT upon addition of OSCP-NT (right axis, top panel, *I*/*I*^0^). *I* and *I*_0_ are the intensities of ^1^H-^15^N cross peaks in the presence or absence of 15-fold molar excess OSCP-NT, respectively. The gray bars on the top of the panel indicate the ^13^Cα secondary chemical shifts (right top axis, in ppm) calculated using the Poulsen library for disordered proteins [72]. Positive secondary chemical shift values are an indication of helical propensity.

To reveal the molecular determinants of the interaction between IF1 and OSCP, we performed NMR titration experiments with the N- and C-terminal (T) domains of both the human proteins. The ^1^H-^15^N SOFAST-HMQC spectra of IF1-NT (G1-E40) and IF1-CT (A44-D81) were chosen to test the interaction with the unlabeled domains of OSCP. The proton signal dispersion in the spectra of IF1-NT (Fig. 3A) and IF1-CT (Fig. S2) is typical of an intrinsically disordered protein and a helical structure, respectively. Comparison of the NMR spectra of ^15^N-labeled IF1-CT upon addition of 12-fold molar excess of OSCP-NT (R6-G114) or OSCP-CT (G114-V190) did not reveal any signal perturbation, nor in terms of chemical shift or line broadening (Fig. S2A and S2B). Then, ^15^N-labeled IF1-NT was titrated using a molar excess (15-fold) of unlabeled OSCP-NT or OSCP-CT. In this case, while the ^1^H-^15^N SOFAST-HMQC spectra of IF1-NT were not perturbed by the presence of OSCP-CT (Fig. S3), a number of peaks were shifted and experienced an attenuated intensity upon addition of OSCP-NT (Fig. 3A). Sequence-specific assignment revealed that the strongest signal perturbations are clustered within a relatively short segment involving about ten amino acids (E29-R39) at the C-terminus of IF1-NT (Fig. 3B), before the coiled-coil domain of the full-length protein. Interestingly, the analysis of secondary chemical shift, indicated that the putative binding region showed the tendency to transiently adopt a helical structure (Fig. 3B). Altogether, these results lead us to the conclusion that the interaction between the two proteins involves a relatively short, disordered segments at the N-terminus of IF1 and the N-terminal domain of OSCP.

**Fig. 4:**
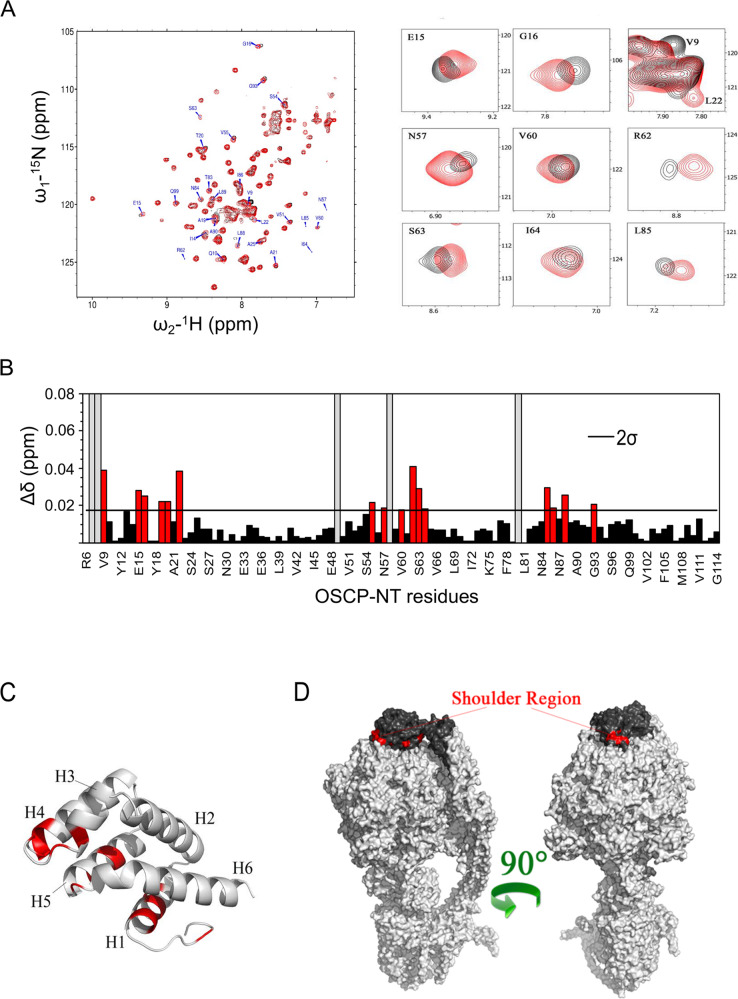
NMR studies revealed the binding region of IF1-NT on a portion of the shoulder region of OSCP, which remains accessible also when OSCP is within the ATP synthase. **A** ^1^H-^15^N SOFAST-HMQC spectrum of OSCP-NT (residues R6-G114) in the absence (red) and in the presence (black) of a 20-fold molar excess of unlabeled IF1-NT (residues G1-E40). The cross peaks with pronounced chemical shift perturbation are labeled and some of them are represented in the expanded plots on the right. **B** Combined chemical shift perturbations derived from the experiments shown in **A**. Red bars indicate residues showing a deviation larger than 2 standard deviations (2*σ*) calculated for all residues. **C** Cartoon representation of OSCP-NT showing in red the residues with the largest chemical shift perturbation (Δδ > 2*σ*). **D** Surface representations of the cryo-EM structure of *Sus scrofa* ATP synthase (PDB code 6J5J) showing two different orientations. The OSCP subunit (both domains) is colored in black. Residues of OSCP-NT affected by the interaction with IF1-NT (with Δδ > 2*σ*) are colored in red. Only residues accessible on the surface of the protein are visible. The shoulder region between helices 3 and 4 is indicated.

To identify the region of OSCP-NT involved in the interaction with IF1, the NMR experiments were repeated monitoring ^1^H-^15^N SOFAST-HMQC spectra of OSCP-NT in the absence and presence of unlabeled IF1-NT (Fig. 4A). The NMR spectrum of OSCP-NT is characterized by relative narrow peaks, well dispersed in both dimensions, compatible with a properly folded structure, as previously reported for the bovine OSCP N-terminal domain [50]. Upon addition of 20-fold molar excess of IF1-NT, a subset of peaks underwent significant shifts and decreased their intensity. Backbone resonance assignment was achieved also for OSCP-NT, and the chemical shift perturbations were mapped on its structure (Fig. 4B and 4C). Remarkably, the larger shifts clustered in two distinct regions of the proteins: helix 1 (H1) and the “shoulder” region comprising helices 3, 4, and 5 (H3, H4 and H5) (Fig. 4C). To verify if these regions are accessible for the interaction with IF1, we considered the OSCP-NT bound with the rest of ATP synthase, using the *Sus scrofa* Cryo-EM structure (PDB code 6J5J [11]). This analysis showed that residues in the “shoulder” region between helices H3 and H4 (A53, S54, N57, V60, R62, S63, and I64) form a continuous area exposed on the surface of the protein (Fig. 4D) available for the interaction with IF1.

These results fully support the IF1-OSCP interaction, revealing its molecular details, and recapitulating the condition of IF1 upregulation in cancer.

### Knocking out and knocking down IF1 decrease colony formation, but do not alter oncogenic/metabolic gene expression

**Fig. 5:**
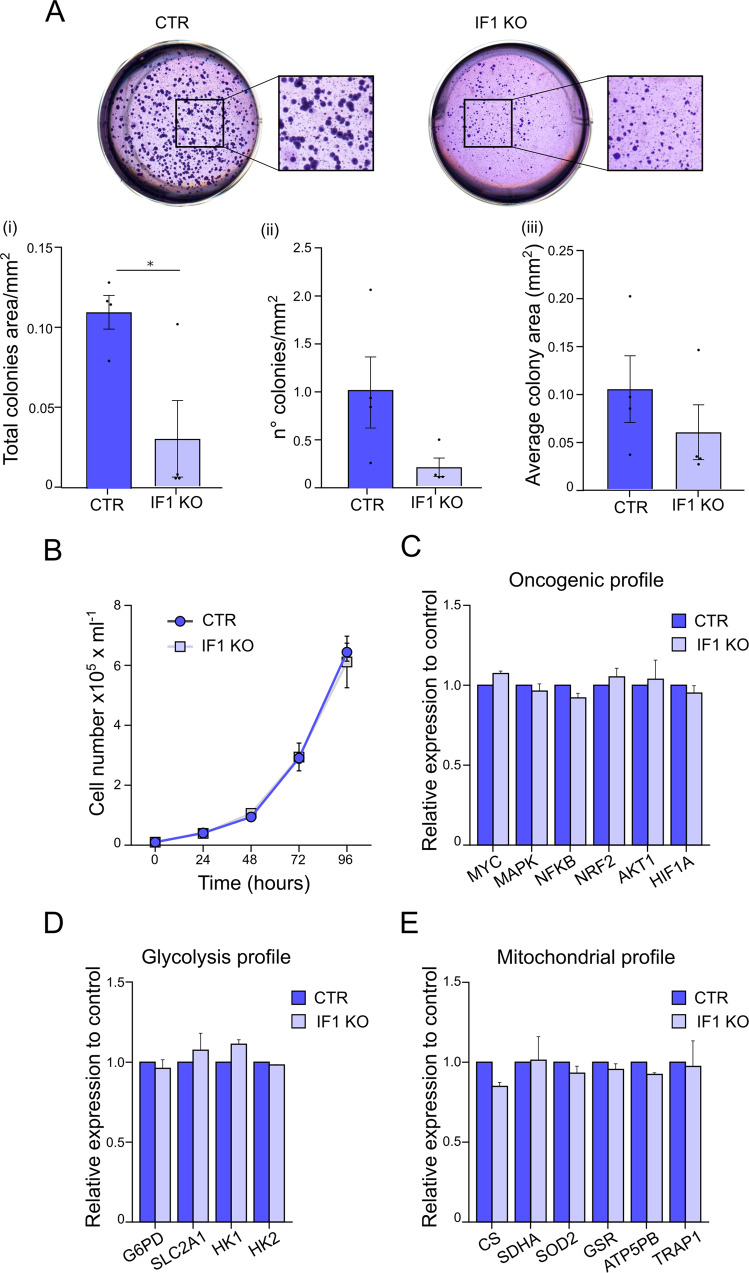
Knocking out the IF1 inhibitor protein in HeLa cells decreases tumor growth in soft agar, but does not alter gene expression of oncogenic/metabolic pathways. **A** Soft agar assay is shown of CTR and IF1 KO HeLa cells. Cells are grown for 15 days and then stained for the quantification analysis. Upper panels, representative images of CTR and IF1 KO cells in stained wells and their magnification. Lower panels, mean of the (i) total colony area/mm^2^, (ii) number of colonies/mm^2^ and (iii) average of colony area in mm^2^. Data are mean ± SEM of four independent experiments, *p = 0.0214. **B** Growth curve of CTR and IF1 KO HeLa cells. Cells are detached and counted every 24 h. Data are mean of three independent experiment ± SEM. Analysis of mRNA relative expression in CTR and IF1 KO HeLa cells. The mRNA levels are analyzed for a selected number of genes involved in **C** oncogenic, **D** glycolytic, and in **E** mitochondrial profiles that are shown. Values are normalized to their levels in CTR and are mean of three independent experiment ± SEM.

To study the role of IF1 in tumor growth, control HeLa cells, and their IF1 KO and IF1 KD counterparts were analyzed for colony-forming capacity in soft agar in vitro (Fig. 5A, S4A). Quantification of the total area occupied by tumor cell colonies, able to grow under stress conditions and insensitive to contact inhibition, resulted significantly higher in IF1- expressing cells (Fig. 5A (i), S4A (i)). In IF KO and IF1 KD cells, fewer and smaller colonies were observed compared to controls (Fig. 5A (ii, iii), S4A (ii, iii)). However, the colony formation promoted by IF1 was not due to increased proliferation in culture (Fig. 5B).

Analysis of expression levels was performed for genes involved in tumor adaptation that might be altered upon selection of IF1 KO and IF1 KD cell populations. In IF1 KO cells, the mRNA level of *MYC*, *MAPK1*, *NFKB1*, *NFE2L2*, *AKT1*, and *HIF1A* genes was unchanged compared to that in controls (Fig. 5C). Genes controlling the glucose uptake and utilization (*G6PD*, *SLC2A1*, *HK1* and *HK2*, Fig. 5D) or mitochondrial function (*CS*, *SDHA*, *SOD2*, *GSR*, *ATP5F1B*, and *TRAP1*, Fig. 5E) were also unchanged. Altered expression was found in IF1 KD cells, expressing 2-fold the levels of *HK1* and *MAPK1*, and 1.5-fold the levels of *AKT1*, of controls (Fig. S4B, S4C). The latest changes might be not biologically relevant since the transcript upregulation of these genes is usually higher in tumors [51–53].

### Knocking out and knocking down IF1 do not affect mitochondrial function, but sensitize mitochondria to permeability transition

**Fig. 6:**
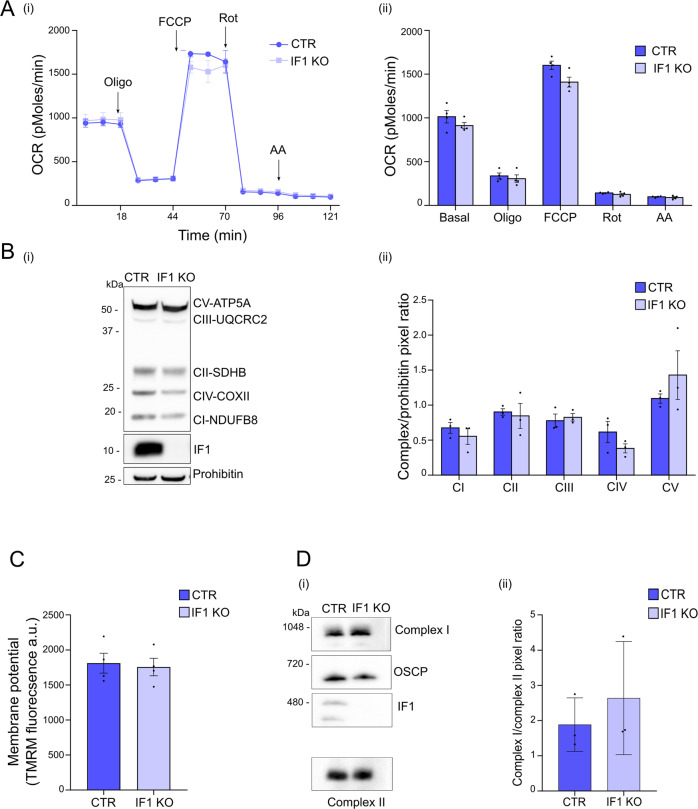
Knocking out the IF1 inhibitor protein in HeLa cells does not affect mitochondrial function. **A** Oxygen consumption rate (OCR) is shown for CTR and IF1 KO HeLa cells. OCR is measured before (basal) and after treatment with oligomycin (oligo), carbonyl cyanide p-(trifluoromethoxy) phenylhydrazone (FCCP), rotenone (Rot) and antimycin A (AA). In (i), a representative OCR measurement of adherent HeLa cells in situ, seeded at the concentration of 50,000 cells/well. In (ii), mean OCR ± SEM of four independent experiments. **B** Western blotting (i) is shown of the indicated OXPHOS complex subunits, IF1 and prohibitin (loading control) in CTR and IF1 KO HeLa cells. The molecular marker is indicated on the left. In (ii), the mean ratio is analyzed of band pixels between each complex subunit and prohibitin (mean ± SEM of three independent experiments). **C** Quantification is shown of TMRM fluorescence monitoring the mitochondrial membrane potential in intact CTR and IF1 KO HeLa cells. Data are expressed in arbitrary units (a.u.) and are mean ± SEM of four independent experiments. **D** Western blotting of BN-PAGE (i) performed on mitochondrial extracts from CTR and IF1 KO HeLa cells (1% w/v digitonin) is shown for NDUFS1 complex I subunit, SDHA complex II subunit, OSCP, and IF1. Molecular markers are on the left. In (ii), the mean ratio (three independent experiments ± SEM) is shown between complex I- containing supercomplexes and complex II band pixels in CTR and IF1 KO cells.

IF1 KO and IF1 KD adherent HeLa cells did not show different mitochondrial respiration than relative controls under basal conditions, upon the addition of the ATP synthase inhibitor oligomycin, the complex I inhibitor rotenone or the complex III inhibitor antimycin A, (Fig. 6A, S4E). In IF1 KO cells, a mild but not significant decrease in oxygen consumption was revealed following the effect of the uncoupler (FCCP, Fig. 6A (ii)) compared to control cells.

Subunits that are important in OXPHOS assembly were analyzed (Fig. 6B). The amount of OXPHOS subunits was unchanged in IF1 KO and control cell lysates as shown by the quantification of their normalized bands, Fig. 6B (ii). The lack of IF1 did not affect the mitochondrial membrane potential in basal condition as measured with TMRM fluorescence (Fig. 6C). Cristae alterations were detected upon IF1 downregulation [24]. Since they were found to affect the respiratory chain complexes in other models [45], super-complex stability was assessed in IF1 KO and control cells by BNE and Western blotting (Fig. 6D), detecting high molecular weight-species containing complex I (Fig. 6D, Complex I), or the OSCP subunit (Fig. 6D, OSCP) to monitor ATP synthase. The results showed that super-complex assembly/stability was unchanged in IF1 KO cells (Fig. 6D).

**Fig. 7:**
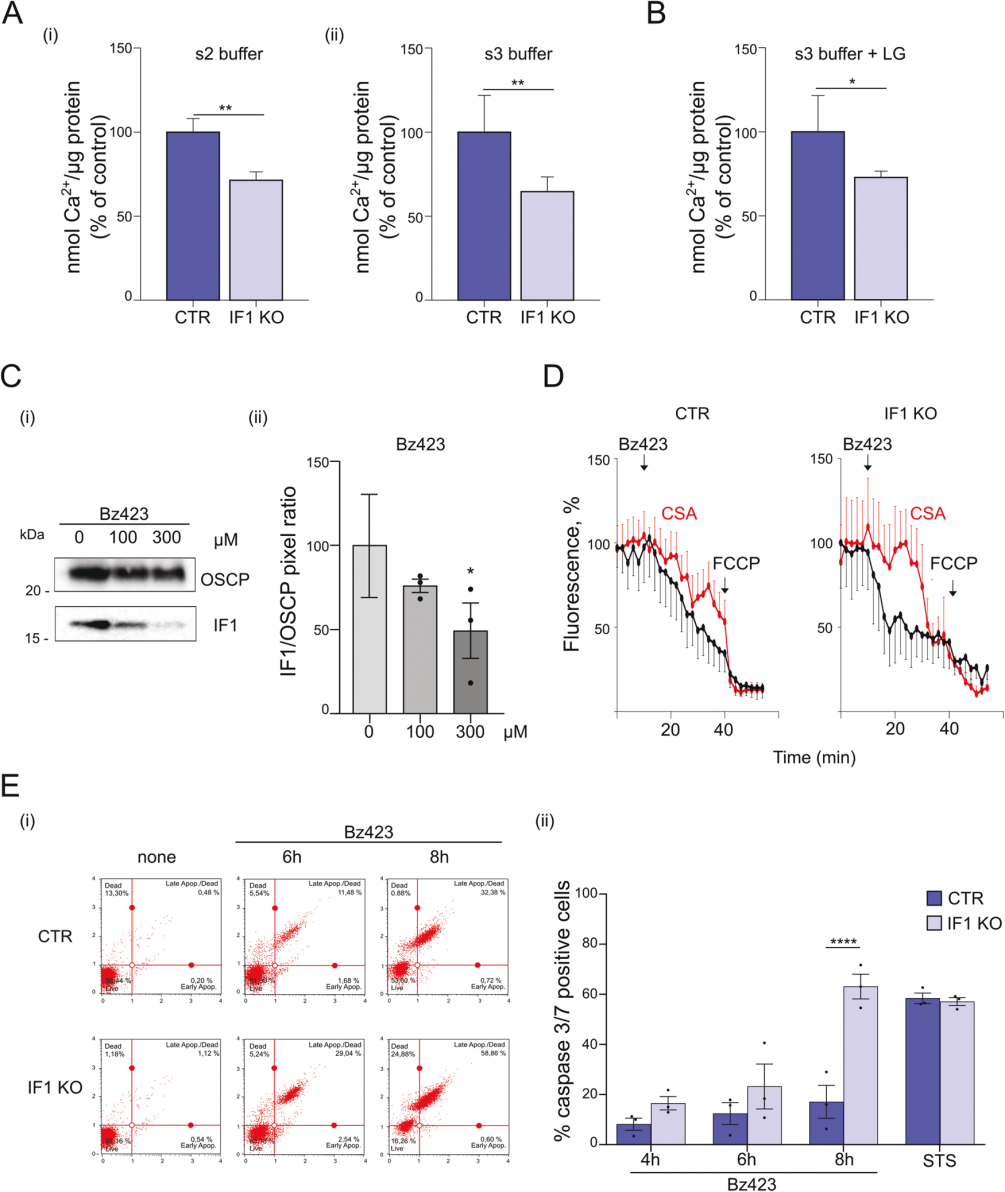
IF1 binding to the OSCP subunit desensitizes respiring HeLa cells to PTP opening and PTP-dependent apoptosis. **A** Calcium retention capacity (CRC) is assessed in CTR and IF1 KO permeabilized HeLa cells in a buffer promoting state 2 (i) or state 3 (ii) respiration and containing the membrane-impermeable Ca^2+^ sensor, Ca^2+^ Green-5N. Histograms represent nmols of Ca^2+^ per µg of protein that are necessary to cause PTP opening. Data are mean (expressed as % of controls) ± SEM of seven or six experiments in (i) or (ii), respectively. *P values* are **p ≤ 0.01. **B** CRC is assessed in CTR and IF1 KO permeabilized HeLa cells in a buffer promoting state 3 respiration and containing the membrane-impermeable Ca^2+^ sensor, Ca^2+^ Green-5N. Cells were starved before the CRC measurements in a 2.5 mM (LG) glucose-containing medium for 24 h. Histograms represent nmols of Ca^2+^ per µg of protein that are necessary to cause PTP opening. Data are mean (expressed as % of controls) ± SEM of three experiments, *p = 0.018. **C** Permeabilized wild-type HeLa cells are incubated in a buffer promoting state 3 respiration, in the presence of the indicated Bz423 concentrations, and processed for immunoprecipitation of OSCP subunit (OSCP). Western blotting (i) is shown to recognize OSCP subunits and dimeric IF1 (indicated on the right). Molecular markers are shown on the left. In (ii), the mean IF1/OSCP pixel ratio is shown (expressed as % of controls) at the different doses of Bz423. Data are from three independent experiments ± SEM, *p = 0.0182. **D** Mitochondrial membrane potential was monitored in intact CTR and IF1 KO HeLa cells, incubated in Ca^2+^-containing HBSS after loading with TMRM. Representative fluorescence traces are shown, in the presence (red) or absence (black) of 1.6 μM CsA. Where indicated 100 μM Bz423 and 4 μM carbonyl cyanide p-(trifluoromethoxy) phenylhydrazone (FCCP) were added. Fluorescence is expressed as % of the initial values. E. Adherent CTR and IF1 KO HeLa cells are treated with 100 μM Bz423 for 4, 6, 8 h, or 2 μM Staurosporine (STS) for 24 h, collected and incubated with a caspase-fluorescent probe, monitoring caspase 3/7 activation, to quantify PTP-dependent apoptotic cells by cytofluorimetric measurements. Representative cytofluorimetric dot-plot profiles (i) of CTR and IF1 KO HeLa cells are shown after 6 h or 8 h of Bz423 treatment or in the absence of treatment (none). In (ii), histograms show the mean ± SEM quantification of apoptotic caspase 3/7-positive CTR and IF1 KO HeLa cells (expressed as % of the total cell population including living and dead cells). Data are from three independent experiments. *P* *value* is ****p < 0.0001.

The analysis of a possible role of IF1 in the modulation of the PTP showed that the Ca^2+^ threshold which promotes PTP opening was significantly decreased in IF1 KO and IF1 KD than in control cells, kept in State 2 and State 3 respiration (Fig. 7A and S4F, respectively). This indicated that IF1 desensitizes respiring cancer HeLa cells to PTP opening, in a mechanism which seems independent of CyPD, as PT is inhibited in both CTR and IF1 KO HeLa cells by the CyPD inhibitor cyclosporin A (CsA) [32] (Fig. S5A). We further tested the role of IF1 on PTP modulation in respiring HeLa cells that were grown in 5 mM glucose-containing medium (LG), to mimic a possible condition of glucose deprivation that might occur in soft agar (Fig. 7B). The Ca^2+^ concentration activating PT was significantly lower in IF1 KO HeLa cells (Fig. 7B), indicating that IF1 inhibits PTP opening in cancer cells under stress conditions.

Our NMR studies indicated that IF1 interacts with the OSCP subunit at the N-terminal shoulder region which is targeted by benzodiazepine (Bz) 423 [54], the known PTP inducer which displaces CyPD [30]. This hypothesis was confirmed when wild-type HeLa respiring cells were treated with increasing doses of Bz423 and subjected to immunoprecipitation for the OSCP subunit (Fig. 7C). The amount of IF1 which immunoprecipitated with the OSCP subunit was displaced by Bz423 in a dose-dependent manner, suggesting a competition for the same binding site. This finding was further supported when the PTP opening was induced in situ by Bz423 treatment and the mitochondrial membrane potential was monitored by tetramethylrhodamine methyl ester (TMRM), comparing CTR and IF1 KO HeLa cells. The drop of membrane potential due to PTP opening was faster in IF1 KO than in CTR-treated cells, in which IF1 masks the Bz423 binding site. Importantly, Bz423-dependent depolarization was inhibited by CsA in both the HeLa cell types (Fig. 7D), confirming the PTP involvement in this mechanism. To test whether IF1 might inhibit PTP-dependent apoptotic cell death, cells were treated with Bz423 in culture conditions. Caspase 3/7-dependent apoptosis was observed 8 h after treatment both in control and IF1 KO HeLa cells, but the presence of IF1 significantly reduced its extent (Fig. 7E and S5B). These findings indicated that IF1 has a role not only in desensitizing the PTP opening but also in protecting cancer cells from PTP- dependent apoptotic death. Staurosporine, a PTP-independent inducer of cell death, equally affected IF1 KO and control HeLa cells (Fig. 7E and S5B), suggesting that IF1 desensitizes apoptosis through the modulation of PTP opening by interacting with the N-terminal part of the OSCP targeted by Bz423.

### The IF1 mutant Y33R binds to OSCP subunit, but not to the F1 sector, causing inhibition of permeability transition and promoting colony formation

**Fig. 8:**
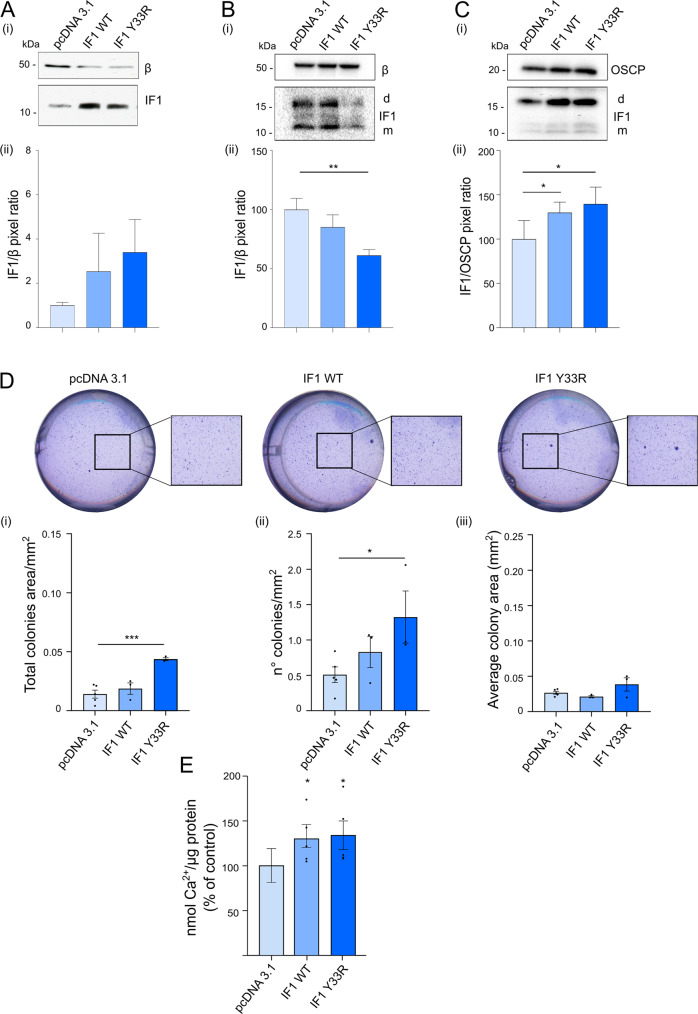
The IF1 mutant Y33R binds to OSCP subunit, but not to the F1 sector, causing inhibition of permeability transition and promoting colony formation. **A** Western blotting is shown of (i) β subunit and IF1 in lysates from mouse embryonic fibroblasts (MEFs) expressing endogenous levels of IF1 (pcDNA3.1, empty vector), or overexpressing the human IF1 either in the wild-type (IF1 WT) or in the mutated (Y33R) form. Molecular markers are on the left. In (ii), histograms of mean IF1/ β subunit pixel ratio are shown (data are mean ± SEM from at least five independent experiments, normalized to pcDNA3.1 set as 1). **B** Permeabilized MEFs described in **A** are incubated in a buffer promoting ATP hydrolysis and processed for immunoprecipitation of β (β) subunit. Western blotting is shown (i) recognizing β subunit and IF1 (indicated on the right; d is dimeric, m is monomeric IF1), representative of three independent experiments. Molecular markers are shown on the left. In (ii), histograms of mean IF1/ β subunit pixel ratio (% of control) are shown (data are mean ± SEM from three independent experiments, **p = 0.0013). Corresponding cell lysates are shown in the original blot file. **C** Permeabilized MEFs described in **A** are incubated in a buffer promoting state 3 respiration and processed for immunoprecipitation of OSCP subunit. Western blotting is shown (i) recognizing OSCP subunit and IF1 (indicated on the right; d is dimeric, m is monomeric IF1), representative of four independent experiments. Molecular markers are shown on the left. In (ii), histograms of mean IF1/ OSCP subunit pixel ratio (% of control) are shown (data are mean ± SEM from four independent experiments, *p ≤ 0.05). Corresponding cell lysates are shown in the original blot file. **D** Soft agar assay is shown of pcDNA3.1, IF1 WT and Y33R MEFs. Cells are grown for 30 days and then stained for the quantification analysis. Upper panels, representative images of pcDNA3.1, IF1 WT, and Y33R MEFs in wells and their magnification. Lower panels, mean of the (i) total colony area/mm^2^, (ii) number of colonies/mm^2^ and (iii) average of colony area in mm^2^. Data are mean ± SEM of at least three independent experiments, ***p = 0.0007; *p = 0.038. **E** Calcium retention capacity (CRC) is assessed in permeabilized pcDNA3.1, IF1 WT, and Y33R MEFs in a buffer promoting state 3 respiration and containing the membrane-impermeable Ca^2+^ sensor, Ca^2+^ Green-5N. Histograms represent nmols of Ca^2+^ per µg of protein that are necessary to cause PTP opening. Data are mean (expressed as % of controls) ± SEM of at least five experiments, *p ≤ 0.05, calculated with the *Student’s* *t-*test between IF1 WT or IF1 Y33R on pcDNA 3.1.

In order to clarify which of the two IF1 binding sites might be involved in the desensitization of the PTP between (1) the canonical site in the F1 sector or (2) the new one on the OSCP subunit, mouse embryonic fibroblasts (MEFs) were generated overexpressing the IF1 mutant Y33R (IF1 Y33R) or the wild-type form of the inhibitor (IF1 WT), Fig. 8A. The Y33R mutant, which introduces a positive-charged arginine, was hypothesized not to bind the F1 catalytic pocket, since in the IF1-F1 bovine crystal structure, residues Y33, L42, and L45 of IF1 are in contact with hydrophobic surfaces [9]. As previously reported, the mutation of IF1 Y33 residue to alanine or tryptophan decreased, or increased, the affinity of the inhibitor for F1-ATPase, respectively. As predicted, the immunoprecipitation for the β subunit, upon ATP hydrolysis, allowed the elution of IF1 in control (pcDNA 3.1, transfection with empty vector) and IF1 WT, but not in IF1 Y33R overexpressing MEFs (Fig. 8B).

On the contrary, the immunoprecipitation of the OSCP subunit, in a condition in which permeabilized MEFs were subjected to active State 3 respiration, allowed the identification of monomeric and dimeric IF1 in the eluted fractions of both IF1 WT and IF1 Y33R overexpressing cells and controls (Fig. 8C). The amount of immunoprecipitated IF1 increased in IF1 WT and IF1 Y33R compared to control MEFs (pcDNA 3.1) of about 30% (Fig. 8C). The selective binding of the IF1 Y33R mutant to the OSCP subunit, but not to the F1 sector, allowed to dissect the two IF1 binding sites and investigate which one is involved in the desensitization of the PTP. The colony-forming capacity in soft agar in vitro was analyzed. The number of colonies and the total colony area were increased upon overexpression of either IF1 WT or IF1 Y33R forms (Fig. 8D). Indeed, this effect was due to the desensitization of the PTP, as seen by direct measurement of the calcium retention capacity in respiring MEFs (Fig. 8E). Both IF1 WT and IF1 Y33R overexpressing MEFs displayed a 30%-higher calcium threshold causing PTP opening during State 3 respiration (Fig. 8E), in line with the 30%-increase in the amount of IF1 which immunoprecipitates with the OSCP subunit in these cells compared to controls (Fig. 8C). This set of experiments allowed to rule out any involvement of the IF1 binding to the F1 sector in the PTP desensitization, since the IF1 Y33R mutant, which is unable to bind the canonical site, caused PTP inhibition in overexpressing MEFs.

## Discussion

This study shows that IF1 interacts with the ATP synthase OSCP subunit in respiring HeLa cells and controls PTP-dependent apoptosis, promoting cancer cell growth.

IF1 is overexpressed in many human tumors [13, 15, 29, 55] and different hypotheses on its pro-oncogenic role have been proposed [13, 16, 29], although its mechanism of action is still debated. MEFs overexpressing IF1, IF1 KD, and IF1 KO HeLa cells showed that colony formation in soft agar depends on IF1 expression level. This result is in line with previous findings showing that IF1 KD decreased colony formation and migration in hepatocellular carcinoma cells [23], and is consistent with the limited colony formation previously reported in IF1 KD HeLa model [26]. The *ATP5IF1* gene ablation supports the above effect of IF1, but also reveals a unique role of this protein in tumorigenic growth, since the contribution of known oncogenic/glycolytic pathways was ruled out by comparing IF1 KO and control HeLa models. Proliferation was unchanged in our IF1-expressing HeLa cells, while their resistance to PT and PTP-dependent apoptosis was higher and sufficient to support their greater propensity to form colonies than the IF1 KO model. The inhibition of ATP synthase by IF1, proposed as the mechanism leading to ROS formation in other cancer models [18], has not been observed in this control HeLa model, as previously reported by Fujikawa and coworkers [56].

Although an increase of the glycolytic flux is often observed in tumors, the OXPHOS can significantly contribute to energy production supporting cell proliferation and metastasis [13, 29, 57, 58]. The finding of a new IF1 binding site on the ATP synthase under active oxidative phosphorylation in cancer cells, controlling PT and resistance to apoptosis, contributes to clarify the role of IF1 overexpression as a pro-survival mechanism in normoxic tumors. In many tumors, pH and O_2_ concentration do not allow the binding of IF1 to the canonical site in the F1 sector of ATP synthase [9, 10]. Immunoprecipitation of the OSCP subunit from both HeLa cells and isolated mitochondria under ATP synthesis conditions showed that the bound IF1 species are mostly dimeric, suggesting that dimers are involved in this interaction. In these forms, the IF1 region E29-R39, involved in the interaction with OSCP, is folded in helical structure [10]. However, these residues are not involved in the coiled coil region and therefore they are potentially available for the interaction with OSCP. The N-terminal region of IF1 presents a remarkable structural plasticity and is prone to interact with different partners depending on the molecular context [59]. In the novel interaction with the OSCP subunit disclosed by our NMR studies, the N-terminal region of IF1 remains largely disordered, but the short segment involved in the interaction presents the tendency to adopt a helical structure even in the isolated protein. This characteristic might be a requisite for the binding with the OSCP subunit, with helical states stabilized in the cellular conditions favoring the interaction.

The IF1 binding to the catalytic β subunit was not detected in immunoprecipitates of respiring cells, confirming that this latter interaction only occurs during ATP hydrolysis [10]. However, both interactions of IF1 with the β and the OSCP subunits were detected in adherent HeLa cells through PLA. This might be explained by transient and heterogeneous mitochondrial depolarization (followed by ATP hydrolysis), as a consequence of multiple factors occurring in situ [60–62].

In our NMR studies, the OSCP residues perturbated by the binding of IF1 are not localized in a single region, but in different sites, including parts of helices H1, H3, H4, H5 and the regions connecting H3–H4 and H5–H6. This observation suggested that the interaction can affect residues not directly involved in the binding epitope, as it was already proposed for Bz423 [54]. The region of OSCP most likely involved in the direct interaction with IF1 is the “shoulder” region comprised between helices H3 and H4 because it was the only perturbed, continuous area, accessible for the binding in the entire ATP synthase structure. Interestingly, this region strongly overlapped with the proposed binding site for Bz423 [54]. This is in good agreement with the experiments in HeLa cells showing that Bz423 displaces IF1 from the OSCP subunit and that Bz423-dependent apoptosis was significantly larger in IF1 KO cells, indicating that IF1 masks the Bz423 site. The inhibition of Bz423-dependent apoptosis is caused by IF1 through PTP desensitization, in line with the role of OSCP subunit in the modulation of PT [30, 31, 42, 44, 63] and the finding that IF1 did not prevent staurosporine-induced cell death in control HeLa cells. The role of IF1 in preventing apoptosis was previously suggested as a consequence of increased cristae density due to enhanced ATP synthase dimer stability [25], which counteracts a Drp1/Bax-dependent apoptotic pathway [24] or an OPA1 processing via OMA1, impeding cristae remodeling [26]. The novel IF1 binding site on the OSCP subunit, although involved in the inhibition of PTP opening, is compatible with other mechanisms inhibiting apoptosis through the maintenance of ATP synthase oligomers and cristae ultrastructure.

Our NMR results indicated that the IF1 interaction-dependent perturbations on the OSCP subunit were revealed in a large molar excess of IF1, in line with the idea that IF1 binding might mostly occur in tumor masses overexpressing the inhibitor protein compared to their control tissues [15]. This effect was recapitulated in our in vivo experiments by injecting in zebrafish embryos HeLa cells, either expressing or not high IF1 levels. Our xenografts show that a high IF1 level correlates with tumor mass development in injected embryos.

The overexpression of IF1 induced a tumor phenotype even in non-tumoral MEF cells, matching a desensitization of PTP opening, previously seen in IF1 overexpressing HEK293 cells [64]. Indeed, the overexpression of the Y33R mutant form of IF1, which cannot bind into the F1 sector, allowed us to figure out the role of the new binding site on the OSCP subunit as the PTP-inhibitor site.

In conclusion, we show that the overexpression of the inhibitor protein IF1 in human tumors may have a double role as a pro-survival mechanism. On the one hand, IF1 inhibits ATP hydrolysis through the canonical binding to the F1 sector, in anoxic and acidic pH conditions; on the other hand, as revealed here, under oxidative phosphorylating conditions, IF1 binds to the ATP synthase OSCP subunit, causing inhibition of PTP opening and preventing apoptosis.

## Materials

Agar, Antimycin A, ATP, ADP, carbonylcyanide-*p*-trifluoromethoxyphenyl hydrazone (FCCP), cyclosporin H, cyclosporin A, digitonin, creatine kinase, EDTA, EGTA, glucose-6-phosphate dehydrogenase, hexokinase, oligomycin, NADP^+^, phosphocreatine, protease inhibitors, pyruvate, rotenone and staurosporine, were from Sigma-Aldrich (St. Louis, MO, USA). Benzodiazepine-423 and digitonin were from Merck (Darmstadt, Germany); TMRM perchlorate was purchased from Molecular Probes (Eugene, OR, USA), Ca^2+^ Green-5N was from Invitrogen (Waltham, MA, USA).

### Experimental model and subject details

HEK 293, HeLa, HepG2, and MEF cells were obtained from the American Tissue Culture Collection (ATCC), Colo741 from the European Collection of Authenticated Cell Cultures (ECACC). Human skin fibroblasts were a kind gift of Prof. Leonardo Salviati.

MEFs and CTR, IF1 KO or IF1 KD HeLa cells were cultured in Dulbecco’s modified Eagle’s medium (DMEM; Lonza, Basel, Switzerland), supplemented with fetal bovine serum (10% v/v), glutamine (2 mM for MEFs or 4 mM for HeLa cells), penicillin and streptomycin (ThermoFisher Scientific, MA, USA). Glucose deprivation assay was obtained by culturing, for 24 h, CTR and IF1 KO HeLa cells in glucose-free DMEM (Thermo Fisher Scientific) supplemented with fetal bovine serum (10% v/v), glutamine (4 mM), penicillin, streptomycin, 2.5 mM glucose, and 5 mM pyruvate. Soft agar assay was performed using phenol red-free DMEM (ThermoFisher Scientific) supplemented with pyruvate (1 mM), penicillin and streptomycin, glutamine (4 mM) and FBS (1% v/v). Fibroblasts were cultured in DMEM, supplemented with fetal bovine serum (20% v/v), glutamine (2 mM), penicillin and streptomycin; HEK293 and HepG2 cells were cultured in DMEM supplemented with fetal bovine serum (10% v/v), glutamine (2 mM), penicillin and streptomycin; and Colo741 cells in RPMI (ThermoFisher Scientific) supplemented with fetal bovine serum (10% v/v), glutamine (2 mM), penicillin and streptomycin. Cells were free of mycoplasma contamination, as routinely tested. Cells were grown at 37 °C in a 5% CO_2_ humidified incubator.

Zebrafish experiments were performed at the Zebrafish Facility of the University of Padova (Italy). Zebrafish embryos were obtained by natural mating of adult fish (Tuebingen line). Embryos and adults were raised and maintained according to standard (ZFIN) protocols. For the injection, the cells (CTR and IF1 KO HeLa) were detached with trypsin, resuspended in serum-containing cell culture medium, washed with PBS, and stained with the fluorescent dye Vybrant DiI (1 mg/mL, catalog number V22885, Thermo Fisher Scientific). The stained cells were injected into zebrafish embryos at 2 days post-fertilization, previously anesthetized with a solution of 160 mg/l tricaine. The cells were injected into the yolk as a single droplet (200 µm diameter, about 100 cells per embryo) using a World Precision Instrument microinjector. One day after injection, the fish were assessed for successful yolk injection and kept in fish water until 4 days post injection. The cell-derived fluorescent tumor mass was imaged at 6 dpf under fluorescence microscopy (M165-FC microscope with DFC7000T digital camera, Leica Microsystems, Milan, Italy) and quantified with the Measurements option of the Volocity 6.0 software (Perkin Elmer, Milan, Italy).

## Methods details

### Stable knocking out of IF1 protein

Stable IF1 knocking out (IF1 KO) was obtained by HeLa transfection with two different guides (G1 and G2) cloned into the lentiCRISPRv2 plasmid and targeting the human IF1 gene. Sequences for the guides (G1: 5′- CGGACGTGGCTTGGCGTGTG -3′ and G2: 5′- CAGTCCGAGAATGTCGACCG -3′) were found by using the CRISPR design tool and were generated following the manufacturer’s procedures. Briefly, oligonucleotide pairs were annealed and cloned into lentiCRISPRv2 plasmid (Addgene, Watertown, MA, USA #52961) and co-transfected into HEK293T cells, with the three packaging plasmids pMDLg/pRRE, pRSV-Rev and pMD2.G, for viral production. HeLa cells were infected by standard methods, with both the recombinant virus containing G1 and with the recombinant virus containing G2. Infected cells were selected with 0.8 μg/ml puromycin and analyzed through western blot. Scrambled lentiCRISPRv2GFP (Addgene #51760) was used as control (CTR).

### Stable knocking down of IF1 protein

Stable interference of IF1 protein (IF1 KD) was obtained by HeLa transfection with a shRNA (sequence: 5′- GATATTTCCGAGCACAGAGTA -3′) cloned into the pLKO.1 lentiviral vector and targeting the human IF1 mRNA. *ATP5IF1* shRNA and its corresponding control (CTR) vector, pLKO.1, were purchased from Sigma (SHC001 Sigma). This shRNA plasmid was co-transfected with the packaging plasmids VSV-G and p8.74 into HEK 293 T cells for viral production. Recombinant virus was collected and used to infect HeLa cells by standard methods. Cells were selected and maintained, in 0.8 µg/ml puromycin for a stable knocking down, and analyzed by Western Blotting. The selected mixed cell populations did not follow single-cell cloning procedure, but IF1 KD cells maintained less than 20% of residual IF1 compared to controls.

### Overexpression of IF1 in MEFs

WT human ATP5IF1 (GenBank NM_016311) was cloned from HeLa cell mRNA by reverse transcription-PCR and inserted into pcDNA3.1(+) (Invitrogen) using the restriction enzymes *HindIII* and *XhoI*. The Y33R mutation (IF1 Y33R) was generated by site-directed mutagenesis using the mutagenic primers:

5′- GAGAGCAGGCTGAAGAGGAACGACGTTTCCGAGC-3′ and

3′-CTCTACTCTGTGCTCGGAAACGTCGTTCCTCTTCAGC-5′, followed by degradation of the original DNA by *DpnI* (New England Biolabs, Ipswich, MA, USA). Mouse Embryonic Fibroblasts (MEFs) were transfected with the empty vector, pcDNA3.1 (pcDNA3.1, control) purchased from Invitrogen (n. V79020), with the pcDNA3.1 containing ATP5IF1 with the mutation Y33R (IF1 Y33R) or with the pcDNA3.1 containing ATP5IF1 WT (IF1 WT) and selected with Geneticin G-418 Sulphate (GIBCO, Life Technologies).

### Bovine IF1 purification

IF1 was purified from bovine heart mitochondria as described in [65]. Mitochondria were heated to 100 °C for 2 min. The suspension was centrifuged at 39 000 x g for 10 min at room temperature, the IF1-containing supernatant was collected while the pellet with all the other denaturized proteins was discarded.

### Lysates, gel electrophoresis, Western blotting

Cells (10 × 10^6^) were kept on ice for 20 min in 0.15 ml of a buffer containing 150 mM NaCl, 20 mM Tris, 5 mM EDTA-Tris, pH 7.4 with the addition of 1% (v/v) Triton X-100, 10% (v/v) glycerol and protease inhibitor cocktail (Merck). Cell extracts were cleared by centrifugation at 18,000 × *g* for 20 min, 4 °C. Sample buffer (NuPAGE™ LDS Sample buffer, Invitrogen) supplemented with 12.5% v/v β-mercaptoethanol) was added to supernatants, and samples were separated by polyacrylamide gel (NuPAGE™, 12% Bis-Tris, Invitrogen) electrophoresis and transferred to nitrocellulose membranes. Blocking was performed with a PBS solution containing 5% (w/v) non-fat dry milk (AppliChem, Darmstadt, Germany). Antibodies for OXPHOS (OXPHOS Human WB Antibody Cocktail), prohibitin, Citrate Synthase, CyPD, IF1, and for β, α, b and OSCP subunits were from Abcam (Cambridge, UK), for γ subunit was from Genetex (Alton Pkwy Irvine CA, USA), for TOM20 was from Santa Cruz Biotechnology (Dallas, TX, USA), and the one against GAPDH was from Cell Signaling (Danvers, MA, USA). Detailed information on the specific antibodies used can be found on the key resource table. Band pixels of each replicate are normalized on band pixels of their proper loading control (β, prohibitin or GAPDH). Mean pixel ratios ± SEM are then shown proportionally to the mean of CTR samples, expressed as 100% or 1.

### Cell permeabilization, mitochondria preparation

For cell permeabilization, cells were detached with trypsin, centrifuged at 1000 × *g* for 5 min, and washed twice with PBS. The pellet was resuspended in KCl-based medium (130 mM KCl, 10 mM Mops-Tris, pH 7.4) supplemented with 1 mM EGTA-Tris and 180 μg/ml digitonin, and incubated for 20 min on ice. Cells were then diluted 1:10 in KCl medium containing 10 μM EGTA-Tris, and centrifuged at 1000 × *g* in a refrigerated centrifuge (4 °C) for 5 min. The final pellet containing permeabilized cells, was used for (i) OSCP and β subunit immunoprecipitation, (ii) oxygen consumption rate and (iii) calcium retention capacity measurements.

Mitochondria from fibroblast, or from HEK, HeLa, Colo741, HepG2 cells were obtained after cell homogenization with a glass-Teflon potter as previously described [66], or in the presence of digitonin (4 mg/ml). In the first case, mitochondria from human cell lines were used for whole ATP synthase or OSCP subunit immunoprecipitation and oxygen consumption rate measurement. In the second case mitochondria prepared with digitonin were used for respiratory chain supercomplex analysis by BN-PAGE.

### Immunoprecipitation of ATP synthase

Immunoprecipitation of ATP synthase was performed in HeLa, Colo741 and HepG2 cells. Briefly, 500 mg of freshly prepared mitochondria were resuspended in 500 µl of a buffer-promoting state 2 respiration (s2: 0.1 M sucrose, 80 mM KCl, 10 mM Mops-Tris, 10 µM EGTA, 5 mM succinate-Tris, 1 mM Pi-Tris, pH 7.4) or state 3 (s3: 0.1 M glucose, 80 mM KCl, 10 mM Mops-Tris, 5 mM succinate-Tris, 4 mM MgCl_2_, 1 mM Pi-Tris, 0.5 mM NADP^+^, 0.4 mM ADP, 50 µM P1, P5-di (adenosine-5′) pentaphosphate, 10 µM EGTA, 4 U/ml glucose-6-phosphate dehydrogenase, and 3 U/ml hexokinase, pH 7.4) respiration, or ATP hydrolysis (H: 0.1 M sucrose, 80 mM KCl, 10 mM Mops-Tris, 4 mM MgCl_2_, 2 mM phosphocreatine, 1 mM Pi-Tris, 0.4 mM ATP, 10 µM EGTA, and 1.5 U/ml creatine kinase, pH 7.4), and incubated 10 min at room temperature. Mitochondria were then centrifuged at 8000 × *g* for 10 min at 4 °C, resuspended in 50 µL of 0.75 M aminocaproic acid, 50 mM Bis-Tris, pH 7.0, and solubilized with digitonin (1% w/v). After centrifugation at 100,000 × *g* for 25 min at 4 °C, the supernatant was collected. Extracted proteins were supplemented with 10 µl of anti-complex V monoclonal antibody covalently linked to protein G-sepharose beads (ATP synthase immunocapture kit, Abcam), and incubated overnight at 4 °C under wheel rotation. Beads were centrifuged at 500 × *g* for 5 min at 4 °C, and washed twice for 5 min in a solution of 0.05% (w/v) n-dodecyl b-D-maltoside (Sigma) in PBS. For the elution, beads were incubated three times with sample buffer 1× for 15 min, and the collected fractions (eluates) were subjected to SDS-PAGE, together with their corresponding digitonin extracts, followed by Western blotting for β subunit and IF1.

### Proximity ligation assay

Proximity ligation assay (PLA) was carried out using the manufacturer’s instructions provided for Duolink In Situ Red Starter kit (DUO92101 Sigma), modified as follows. Sterile coverslips were placed in a 24-well plate. IF1 KO and CTR HeLa cells were seeded at 50 × 10^3^ cells/well on coverslips and after growing for 24 h fixed with 2% (w/v) paraformaldehyde, permeabilized with 0,15% (v/v) Triton X-100 for 5 min at room temperature and washed with PBS. Cells were incubated with Duolink Blocking Solution for 30 min at room temperature under gentle shacking. According to the manufacturer’s instructions, two primary antibodies were used in combination in each coverslip, in order to detect possible interactions occurring between two proteins in close proximity. The primary antibodies applied in couple derived from different species (i.e., mouse (M) or rabbit (R)) and were: anti-ATPB ab14730; anti-ATP5A ab110273; anti-ATPase IF1 ab223779; anti-ATPase IF1 ab110277; anti-Cyclophilin F ab110324; anti-ATP5O ab110276; anti-OSCP SC74786; anti-c ab1181243; anti-f ab200715, diluted 1:30 in Duolink Antibody Diluent for incubation overnight, at 4 °C into a humidity chamber. The day after, secondary antibodies conjugated with oligonucleotides (PLA probe anti-mouse MINUS and PLA probe anti-rabbit PLUS) were incubated with samples for 1.5 h at 37 °C. Ligation and amplification were performed according to manufacturer’s instructions. The amplification reaction mix was incubated for 2 h at 37 °C in the humidity chamber. After washing, coverslips were mounted with Duolink In Situ Mounting Medium containing DAPI fluorescent probe for nuclei detection. Red dots detecting protein interactions were analyzed through a Leica TCS SP5 confocal microscope equipped with a CCD camera and a ×40 objective.

### Immunoprecipitation of OSCP or β subunit

Immunoprecipitation of OSCP or β subunit was carried out starting from (i) freshly prepared HeLa mitochondria or from (ii) permeabilized MEFs or HeLa cells. Briefly, 400 µg of mitochondria or 15 millions of permeabilized cells, were resuspended in 400 µl or in 750 µl, respectively, of a medium promoting ATP synthesis, or ATP hydrolysis, as previously described, and incubated for 10 min at room temperature. Mitochondria, or permeabilized cells, were then precipitated (8000 × *g* for 10 min at 4 °C or 1000 × *g* for 5 min at 4 °C, respectively), resuspended in 400 µl of the IP buffer (50 mM NaCl, 30 mM Tris-HCl, 2 mM EGTA-Tris, 5 mM aminocaproic acid, 0.005% (w/v) BSA, 0.5% (v/v) Triton X-100, 0.25% (w/v) SDS, pH 7.4), supplemented with 2 µg of anti- OSCP or anti- β antibody (Abcam) and incubated for 2 h at 4 °C under wheel rotation. Finally, 30 µL dynabeads protein G (Invitrogen) were added, and samples were incubated overnight at 4 °C under wheel rotation. The day after, the supernatant was collected in a clean tube, beads were washes three times with IP buffer devoid of SDS and proteins were eluted with sample buffer 1X. The collected fractions were subjected to SDS-PAGE followed by Western blotting to detect OSCP or β subunit, and IF1.

### Protein expression and purification

DNA fragments encoding for the different constructs of human OSCP (UniProtKB-P48047) and human IF1, isoform 1 (UniProtKB-Q9UII2) were cloned in Champion^TM^ pET SUMO expression vector and the plasmids were used to transform competent *E. coli* BL21(DE3). Cells were grown at 37 °C, 220 rpm up to an OD-600 nm of 0.6–0.8 and protein expression induced with 0.5 mM IPTG at 20 °C, 220 rpm for 20 h. Unlabeled protein was expressed in LB medium, while uniformly ^15^N or ^15^N/^13^C doubly-labeled proteins were produced in M9 minimal medium supplemented with 1 g/L ^15^NH_4_Cl and 4 g/L glucose or ^13^C-glucose, respectively. Kanamycin (50 µg/ml) was added as selection agent. Cells were harvested by centrifugation (5000 × g, 20 min, 4 °C). Bacterial pellets were resuspended in 50 mM sodium phosphate, 300 mM NaCl, pH 7.8 (buffer A) supplemented with 10 mM imidazole, 60 μM PMSF, Complete^TM^ EDTA-free protease inhibitor tablet (Roche, Basel, Switzerland), bovine DNAase I and 1 mg/ml MgCl_2_. The cells were broken by passing them twice through a French press. The lysates were cleared by centrifugation (12,000 × g for 30 min at 4 °C) before proceeding with purification as described in details below for the different proteins.

In all cases the proteins of interest were expressed fused with a N-terminal His6SUMO-tag, which was removed with His6-Ulp1 protease at mass ratio 1:100 (enzyme:protein), leaving untagged proteins without additional residues. The purified proteins were flash-frozen and stored at – 80 °C.

#### OSCP-NT (R6-G114)

The cleared lysate was loaded onto a 5 ml Ni^2+^-HisTrap^TM^ FF column equilibrated in buffer A with 10 mM imidazole. His6SUMO-OSCP-NT was eluted with 250 mM imidazole in buffer A. Imidazole was removed with a HiPrep^TM^ 26/10 desalting column equilibrated in buffer A. The protein was concentrated to 5 ml by ultracentrifugation (Vivaspin 5 kDa MWCO, 5500 × g × 15 min, 4 °C) and then incubated at 15 °C overnight with His6-Ulp1. The cleaved protein sample was diluted with 50 mM sodium phosphate buffer (pH 7.0) to reach a final NaCl concentration of 150 mM, and then pH was adjusted to a value of 7.0. The sample was loaded onto a 1 ml HiTrap^TM^ SP FF cation exchange column equilibrated in 50 mM sodium phosphate, 150 mM NaCl, pH 7 (buffer B). After washing the column with buffer B, OSCP-NT was eluted from the column increasing NaCl concentration up to 500 mM and finally applied to Superdex 75 PG HiLoad 16/60 column equilibrated in 50 mM phosphate, 300 mM NaCl, pH 7 (buffer C). The doubly labeled protein was equilibrated in 50 mM phosphate, 200 mM NaCl, 50 mM L-glutamic acid, 50 mM L-arginine, pH 7.

#### OSCP-CT (G114-V190)

His6-SUMO-OSCP-CT was purified from *E. coli* lysate as previously described for His6SUMO-OSCP-NT but using 5 mM β-mercaptoethanol (β-ME) in buffer A. The enzymatic cleavage was performed at 4 °C overnight. The cleaved protein was loaded to a 5 ml Ni^2+^ - HisTrap^TM^ FF column equilibrated in buffer A with 5 mM β-ME and the flow-through containing human OSCP-CT was collected. OSCP-CT was applied to a Superdex 75 PG HiLoad 16/60 column (GE Healthcare, Chicago, IL, USA) equilibrated in buffer C. Purified fractions containing OSCP-CT were collected and concentrated as described before. DTT (2 mM) was added to OSCP-CT before concentration.

#### IF1-NT (G1-E40)

His6SUMO-IF1-1NT was purified from *E. coli* lysate as previously described for His6SUMO-OSCP-NT but no imidazole was added to buffer A in this case. Enzymatic digestion with His6-Ulp1 was performed at 4 °C overnight. After cleavage, IF1-1NT was separated from His6-SUMO, His6-Ulp1 and other contaminants as previously described for OSCP-CT. Fractions containing target protein were loaded onto a Superdex 75 PG HiLoad 16/60 column equilibrated in buffer C. Purified fractions containing IF1-NT were collected and concentrated by ultracentrifugation (5500 × g ×15 min, 4 °C). ^13^C/^15^N-doubly labeled IF1-NT was purified as for the unlabeled protein but the final SEC column was equilibrated in 50 mM phosphate, 50 mM L-glutamic acid, 50 mM L-arginine, pH 7.

#### IF1-CT (A44-D81)

The soluble lysate was loaded onto a 5 ml Ni^2+^ - HisTrapTM FF column equilibrated in buffer A and the flow through was discharged. His6-SUMO-IF1-1CT was eluted with other contaminants increasing the imidazole concentration up to 250 mM. Then, the imidazole was removed with a HiPrep^TM^ 26/10 desalting column equilibrated in 20 mM sodium phosphate, 150 mM NaCl, pH 7.8 (buffer D). The eluted fractions were pooled and concentrated as described previously. Enzymatic digestion was performed at room temperature overnight. The cleaved protein was diluted with 20 mM sodium phosphate buffer (pH 7.5) to reach a final NaCl concentration of 25 mM and then pH was adjusted to a value of 7.5. The sample was then loaded onto a 1 ml HiTrap^TM^ SP FF cation exchange column equilibrated in 20 mM phosphate at pH 7.5 (buffer E) and the flow through containing His6-SUMO and other contaminants was discharged whereas IF1-CT was eluted from the column increasing NaCl concentration up to 250 mM. IF1-CT fractions were pooled and then applied to a Superdex 75 PG HiLoad 16/60 column equilibrated in buffer C. Purified fractions containing IF1-CT were pooled and concentrated as described before. ^15^N- labeled IF1-CT was purified for the unlabeled protein.

### Nuclear magnetic resonance (NMR)

All NMR experiments were performed with a Bruker AVANCE NEO 600 MHz spectrometer, equipped with a 5 mm cryogenic probe Prodigy TCI. All NMR spectra were processed with Bruker Topspin 4.0.6 (Bruker BioSpin GmbH, Rheinstetten, Germany) and analyzed using CARA 1.9 [67] and NMRFAM-Sparky [68].

#### Backbone assignment experiments

Experiments for OSCP-NT were performed on a sample of 0.5 mM uniformly ^13^C/^15^N-labeled protein in 50 mM phosphate, 200 mM NaCl, 50 mM L-glutamic acid, 50 mM L-arginine, pH 7 at 25 °C. Experiments for IF1-NT were performed on a sample of 1.3 mM uniformly ^13^C/^15^N-labeled protein in 50 mM phosphate, 50 mM L-glutamic acid, 50 mM L-arginine, pH 7 at 7 °C. Final protein samples were prepared by adding D_2_O for a total concentration of about 4% (v/v), 1 mM EDTA, 60 μM PMSF, Complete^TM^ EDTA-free protease inhibitor tablet (Roche) and NaN_3_ 0.05% (w/v). Around 300 μl of each sample were loaded into the Shigemi microtubes (Shigemi Inc, PA, USA) for data collection.

Sequence-specific backbone assignments of OSCP-NT and IF1-NT were achieved using 2D 1H-15N HSQC, 3D HNCA, 3D HN(CO)CA, 3D HNCO, 3D HN(CA)CO, 3D CBCA(CO)NH and 3D HNCACB. All triple resonance experiments were implemented with the “best” approach [69] using Bruker Topspin 4.0.6 pulse programs with default parameter sets (b_hncagp3d.2, b_hncocagp3d.2, b_hncogp3d.2, b_hncacogp3d.2, b_hncacbgp3d.2, b_nhnocacbgp3d.2). Experimental sweep widths, acquisition times, and the number of transients were optimized for the necessary resolution, experiment time, and signal to noise for each experiment type.

#### Interaction experiments

To study the interaction between OSCP and IF1 domains by NMR, ^1^H- ^15^N SOFAST HMQC spectra [70] were acquired on ^15^N -labeled domains (30–100 μM) with and without the unlabeled partner in large molar excess (12-20X).

Final experiments to characterize the interaction between OSCP-NT and IF1-NT, were acquired on the following samples:

- 30 μM ^15^N-labeled IF1-NT, in the absence and in presence of unlabeled 450 μM OSCP-NT, in buffer containing 50 mM phosphate, 300 mM NaCl, D_2_O 4% (v/v), pH 7.0, at 7 °C.
- 100 μM ^15^N-labeled OSCP-NT, in the absence and in presence of unlabeled 2 mM IF1-NT, in buffer containing 50 mM phosphate, 80 mM NaCl, D_2_O 4% (v/v), pH 7.0, at 25 °C.

Chemical shifts perturbation (Δδ) for each cross-peak was calculated using the following equation:

$$\Delta \delta = \sqrt {\frac{{(\Delta \delta {\it{H}})^2 + \left( {\frac{{\Delta \delta {\it{N}}}}{5}} \right)^2}}{2}}$$

where ΔδH and ΔδN represent the ^1^H and ^15^N amide chemical shift changes respectively. We used the standard deviation *σ*, calculated across the whole sequence, as the threshold value: residues with Δ*δ* > 2*σ* were considered significantly perturbed. The significantly perturbed residues were mapped on OSCP-NT by using a model of the human domain (6–114) predicted by AlphaFold [71] and the Cryo-EM structure of *Sus scrofa* ATP synthase (PDB code 6J5J). All structures were visualized using Pymol.

To evaluate the residue-specific, secondary structure propensity of the disordered IF1-NT, we calculated the secondary chemical shift for all ^13^Cα atoms, using the Poulsen library [72] for disordered proteins: positive secondary chemical shift values are diagnostic of helical propensity.

### Soft Agar assay

For the soft agar assay a phenol red-free DMEM medium, supplemented with glutamine (4 mM), pyruvate (1 mM), penicillin and streptomycin were used.

HeLa cells (IF1 KO or IF1 KD and controls) were seeded at 25,000 cells/well in a 12-well tissue culture plate in a 0.6% (w/v) agar matrix, between a bottom layer composed of 1% (w/v) agar matrix and a top layer of complete DMEM supplemented with 1% v/v FBS. MEFs were seeded at 54,000 cells/well and DMEM supplemented with 4% FBS. HeLa and MEF cells were grown 15 or 30 days, respectively at 37 °C in a 5% CO_2_ humidified incubator and the upper layer medium was changed every 2 days. After 15 or 30 days, colonies were washed in PBS, stained with a 0.005 (w/v) Crystal Violet solution (Sigma) and analyzed with ImageJ software.

### Growth curve

HeLa cells (CTR and IF1 KO) were seeded at 10,000 cells/well in a 12 well tissue culture plate in DMEM containing 25 mM glucose. Cells were incubated at 37 °C in a 5% CO_2_ humidified incubator and cultured for 96 h. After 24, 48, 72, and 96 h cells were harvested with trypsin and counted.

### Real-time PCR

#### RNA extraction and cDNA synthesis

Total RNA was extracted using TRIzol™ Reagent (Invitrogen™), resuspended in Sterile nuclease-free DEPC water and treated with RNase-free DNase I (Promega, Madison, WI, USA) to eliminate genomic DNA contamination. Quality and quantity of RNA were evaluated using NanoDrop™ 2000/2000c Spectrophotometer (ThermoFisher Scientific). cDNA synthesis reactions were performed with 6 μg of total RNA and Random Hexamer using the SuperScript™ III First-Strand Synthesis System (Invitrogen) according to manufacturer’s instructions.

#### Primer design and validation

A primer set of 16 key genes related with different pathways was designed using Primer3 v.4.1.0. The criteria included were primer design in the exonic region of each gene and PCR products between 90 and 250 bp in size. A list of genes and primers is summarized in Supplementary table 1. The amplification efficiency of each primer pair was calculated based on the standard curve generated from a two-fold dilution series of cDNA spanning five orders of magnitude (36 pg ~ 100 ng). Slope of the standard curve was used to measure the amplification efficiency of a qPCR reaction (qPCR Efficiency Calculator -ThermoFisher). The primer amplification efficiency was corroborated for each sample and condition. Moreover, *GUSB* was selected as a reference gene for expression analysis due to the levels of expression being stable in HeLa cells under different metabolic conditions [73].

#### Quantitative real-time PCR and expression analysis

qPCR assays were performed using CFX96 Touch Deep Well Real-Time PCR Detection System (BioRad, Hercules, CA, USA). Amplifications were carried out with 150 ng of cDNA from each sample by using GoTaq qPCR Master Mix (Promega). To check reproducibility, each assay was performed three times with technical triplicates for each sample. PCR efficiency values (*E*) were calculated for each gene and cell culture from the given slope after running standard curves and following the formula *E* = (10^(−1/slope)^−1) × 100

Crossing threshold (Ct) values were determined by CFX Maestro Software (BioRad) and relative expression data were analyzed by the Individual Efficiency Corrected Calculation Method based on the 2^−ΔΔCT^ method [74]. Finally, data were normalized by the expression values of HeLa controls in each condition.

#### Oxygen consumption

Oxygen consumption rate was measured in freshly prepared HeLa mitochondria, or permeabilized HeLa cells, at 30 °C with 20 mM succinate as substrate, using an oxygen Clark-type electrode. State 3, state 4 and uncoupled respiration rates were measured after the addition of 0.5 mM ADP, 0.75 μM oligomycin, or 0.2 μM FCCP, respectively.

Oxygen consumption rate (OCR) in adherent cells was measured using the XF24 Extracellular Flux Analyzer (Agilent technologies, Santa Clara, CA, USA) [75, 76]. Briefly HeLa cells were seeded in XF24 cell culture microplates at 50,000 (CTR, IF1 KO or IF1 KD) cells/well and let grow at 37 °C in a 5% CO_2_ humidified incubator for 24 h. The day after, the growth medium was replaced with the Seahorse medium (DMEM, Sigma D5030, supplemented with NaCl, glutamine and phenol red according to the manufacturer protocol and 25 mM glucose, 10 mM sodium pyruvate), and cells were incubated at 37 °C for 30 min to allow temperature and pH equilibration. After an OCR baseline measurement, 1 mg/ml oligomycin, 0.1 mM FCCP, 1 mM rotenone, and 1 mM antimycin A were sequentially added to each well. Before each experiment, a titration with FCCP was performed to determine the optimal FCCP concentration that stimulates respiration maximally, which was found to be 0.1 mM for all cell types.

#### Mitochondrial membrane potential

Mitochondrial membrane potential was measured based on the mitochondrial accumulation of TMRM in intact cells. HeLa cells (CTR, IF1 KO) were seeded at 30000 cells/well in a 12-well tissue culture plate. The day after seeding, cells were incubated for 30 min at 37 °C in an FBS free-DMEM medium containing 20 nM TMRM and 1.6 μM cyclosporin H to inhibit the multidrug resistance pump. Cells were then washed, detached with trypsin, centrifuged at 1000 × g for 5 min, and suspended in PBS. Mitochondrial membrane potential was immediately analyzed by flow cytometry using the Muse cell analyzer (Millipore, Burlington, MA, USA). Data acquisition and analysis were performed with MuseSoft Analysis and Flowing software, respectively. A total of 5000 events were acquired for each determination. Mitochondrial membrane potential was monitored based on mitochondrial accumulation of TMRM by epifluorescence microscopy, and time-dependent depolarization was analyzed as previously reported [75, 76], upon addition of 100 μM Bz423 and 4 μM FCCP.

#### Blue native PAGE

For the respiratory chain supercomplex analysis, mitochondria were resuspended in 100 μl of a buffer composed of 1.5 M aminocaproic acid, 50 mM Bis-Tris, pH 7.0, solubilized with 1% (w/v) digitonin, incubated on ice for 5 min and solubilized protein complexes were separated through centrifugation at 20,000 × *g* for 30 min at 4 °C.

Supernatants were collected, supplemented with a sample buffer prepared with 5% (w/v) Serva Blue G, 1.0 M aminocaproic acid and quickly loaded onto a 3–12% blue native polyacrylamide gradient gel (BN-PAGE, Invitrogen). After electrophoresis, gels were used for Western Blotting against Ndufs1 (Complex I, Abcam), OSCP, IF1 or SDHA (Complex II, Santa Cruz Biotechnology) subunits.

#### Ca^2+^ retention capacity

For the Calcium Retention Capacity (CRC) assay, external mitochondrial Ca^2+^ was measured by Ca^2+^ Green-5N fluorescence using a Tecan Infinite® 200 PRO (Tecan Trading AG, Switzerland) plate reader. IF1 KO, IF1 KD and control HeLa cells, or MEFs, were permeabilized as mentioned above and were resuspended, at the concentration of 10^6^ × ml^−1^, in a buffer promoting state 2 (s2: 0.1 M sucrose, 80 mM KCl, 10 mM Mops-Tris, 10 µM EGTA, pH 7.4) or state 3 (s3: 0.1 M glucose, 80 mM KCl, 10 mM Mops-Tris, 4 mM MgCl_2_, 0.5 mM NADP^+^, 0.4 mM ADP, 50 µM P1, P5-di(adenosine-5′) pentaphosphate, 10 µM EGTA, 4 U/ml glucose-6-phosphate dehydrogenase, and 3 U/ml hexokinase, pH 7.4) respiration, supplemented with 5 mM succinate-Tris, 1 mM Pi-Tris and 0.5 μM Ca^2+^ Green-5N to a final volume of 0.2 ml. For glucose deprivation studies (LG), CTR and IF1 KO HeLa cells were kept with 2.5 mM glucose for 24 h, and then the CRC assay was performed as mentioned above. For all CRC measurements, sequential 5 μM CaCl_2_ pulses were added to cells and Ca^2+^ Green-5N fluorescence was measured.

#### Cell death

HeLa cells (IF1 KO and CTR) were seeded at 500,000 cells/well in a six wells tissue culture plate. The day after seeding, cells were incubated with 100 μM Bz423 in bicarbonate- and phenol red-free Hanks’ balanced salt solution (HBSS) containing 10 mM HEPES, pH 7.4 at 37 °C. After 4, 6, 8 h of treatment, cells were harvested with trypsin and counted. Cells incubated only with HBSS, containing 10 mM HEPES pH7.4, at 37 °C for 8 h were harvested with trypsin and counted, for control. Staurosporine (2 μM) treatment was used as positive control for cell death, and was incubated in the same conditions above for 24 h. Cell death was assessed by the Muse cell analyzer (Millipore) using Muse Caspase - 3/7 Kit (Luminex Flow Citometry & Imaging), or Muse Annexin V and Dead Cell Kit (Luminex Flow Cytometry & Imaging), following the manufacturer’s instructions.

#### Quantification and statistical analysis

Unless otherwise stated in the figure legends, each experiment was repeated at least three times. Data are presented as mean ± SEM. *P* values indicated in the figures are calculated with GraphPad, Student’s *t* test is applied (∗represents *p* ≤ 0.05, ∗∗*p* ≤ 0.01, ∗∗∗*p* ≤ 0.001), or *one/two-way* ANOVA. The variance between the groups that are compared is similar. Western blotting band intensities were analyzed using ChemiDoc MP system equipped with the ImageLab software (Bio-Rad) or ImageJ software, while TMRM and cell apoptosis measurements were analyzed using the Muse cell analyzer (Millipore) and MuseSoft Analysis and Flowing software.

## Supplementary information

Figure S1

Figure S2

Figure S3

Figure S4

Figure S5

Table S1

Original blot

author checklist

## Data Availability

All constructs will be made available to the scientific community upon request.
