# Supplementary material for: The mitochondrial inhibitor IF1 binds to the ATP synthase OSCP subunit and protects cancer cells from apoptosis
Source: Cell Death Dis. 2023 Jan 23;14(1):54. doi: 10.1038/s41419-023-05572-y (PMC9870916; doi:10.1038/s41419-023-05572-y)
Supplement: Supplementary file 1 — Figure S1 [file 41419_2023_5572_MOESM1_ESM.pdf]

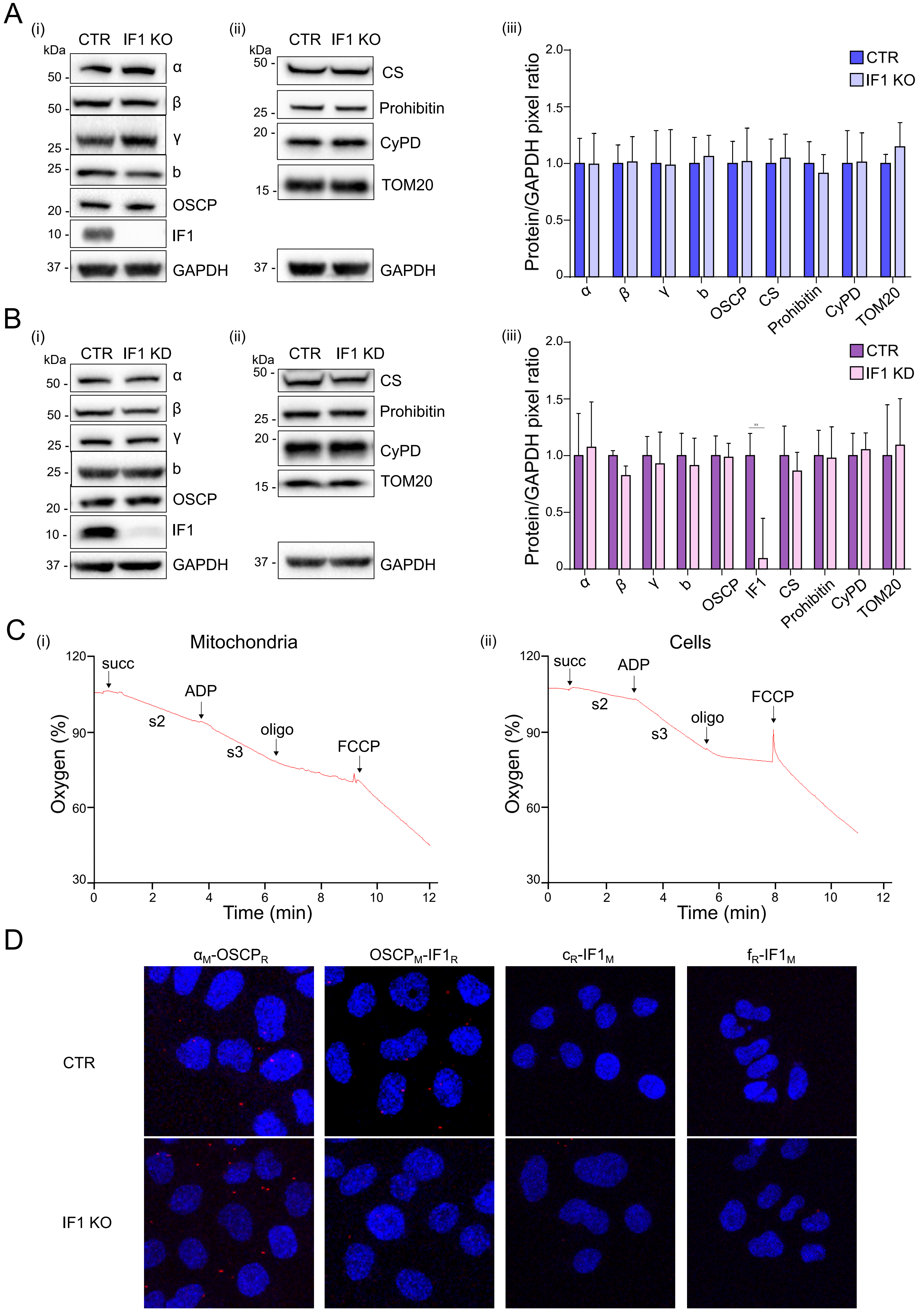
A
(i)
CTR IF1 KO
kDa
α
β
γ
b
OSCP
IF1
GAPDH
(ii)
CS
Prohibitin
CyPD
TOM20
GAPDH
(iii)
Protein/GAPDH pixel ratio
CTR
IF1 KO
B
CTR IF1 KD
IF1 KD
C
Mitochondria
Cells
succ
ADP
oligo
FCCP
s2
s3
Oxygen (%)
Time (min)
D
αM-OSCPR
OSCPM-IF1R
cR-IF1M
fR-IF1M
CTR
IF1 KO

Figure S1

A. Western blotting is shown of the ATP synthase α, β, γ, b, OSCP subunits and IF1 (i) or citrate synthase (CS), prohibitin, CyPD, TOM20 (ii) in CTR and IF1 KO HeLa cell lysates. Glyceraldehyde 3-phosphate dehydrogenase (GAPDH) is detected in (i) and (ii) as loading control. Molecular markers are on the left. Mean ratio of at least 4 independent experiment ± SEM is shown (iii) between each protein detected and GAPDH band pixels (relative to the ratio in controls) in CTR and IF1 KO HeLa cells.

B. Western blotting is shown of the ATP synthase α, β, γ, b, OSCP subunits and IF1 (i) or citrate synthase (CS), prohibitin, CyPD, TOM20 (ii) in CTR and IF1 KD HeLa cell lysates. Glyceraldehyde 3-phosphate dehydrogenase (GAPDH) is detected in (i) and (ii) as loading control. Molecular markers are on the left. Mean ratio of 4 independent experiment ± SEM is shown (iii) between each protein detected and GAPDH band pixels (relative to the ratio in controls) in CTR and IF1 KD HeLa cells. *P value* is **p= 0.0038.

C. Complex II-driven oxygen consumption rate (state 2 respiration, s2) is measured in HeLa mitochondria (i), or HeLa permeabilized cells (ii) upon addition of 5 mM succinate. ADP, oligomycin (oligo) and FCCP were added in the chamber to measure, state 3 (s3), state 4 and FCCP-stimulated respiration. Additions are indicated with arrows on each trace, representative of 3 independent experiments.

D. Representative images of CTR and IF1 KO HeLa cells processed using the Proximity Ligation Assay (PLA) to assess protein-protein interactions. The antibodies used in combination to test the interactions between mitochondrial proteins are indicated and detect the α (α), OSCP (OSCP), c, f subunits and IF1 (IF1). M or R indicate the secondary antibodies used during the PLA protocol. Interactions are revealed by red dots, while DAPI-stained nuclei are in blue. Images are acquired with a Leica TCS SP5 confocal microscope equipped with a CCD camera and a 40x objective.
