# Supplementary material for: The mitochondrial inhibitor IF1 binds to the ATP synthase OSCP subunit and protects cancer cells from apoptosis
Source: Cell Death Dis. 2023 Jan 23;14(1):54. doi: 10.1038/s41419-023-05572-y (PMC9870916; doi:10.1038/s41419-023-05572-y)
Supplement: Supplementary file 2 — Figure S2 [file 41419_2023_5572_MOESM2_ESM.pdf]

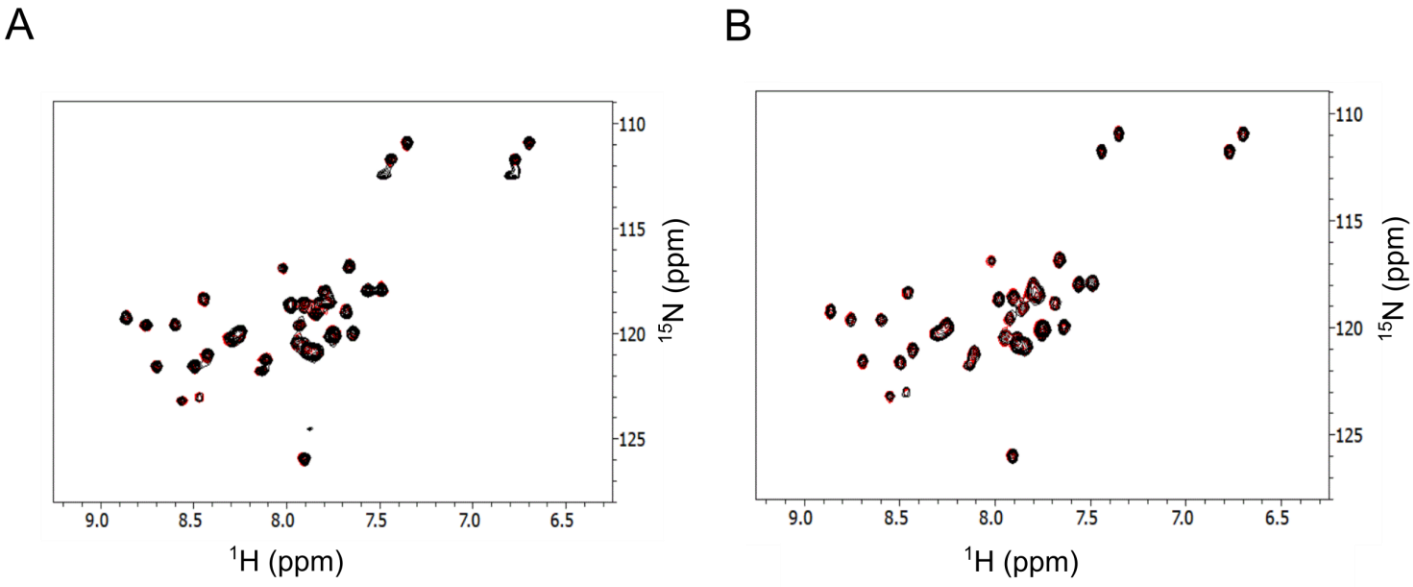

Figure S2

A. Superimposition between the ^{1}H-^{15}N SOFAST HMQC spectra of ^{15}N-labelled IF1-CT at final protein concentration of 40 μM in the absence (red spectra) and presence (black spectra) of 480 μM unlabelled OSCP-CT.

B. Superimposition between the ^{1}H-^{15}N SOFAST HMQC spectra of ^{15}N-labelled IF1-CT at final protein concentration of 40 μM in the absence (red spectra) and presence (black spectra) of 480 μM unlabelled OSCP-NT.
