# Supplementary material for: The mitochondrial inhibitor IF1 binds to the ATP synthase OSCP subunit and protects cancer cells from apoptosis
Source: Cell Death Dis. 2023 Jan 23;14(1):54. doi: 10.1038/s41419-023-05572-y (PMC9870916; doi:10.1038/s41419-023-05572-y)
Supplement: Supplementary file 3 — Figure S3 [file 41419_2023_5572_MOESM3_ESM.pdf]

A

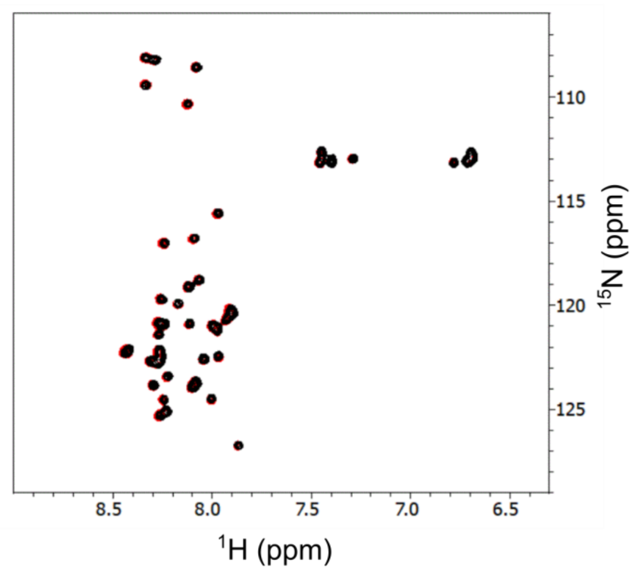


**Figure S3**

A. Superimposition between the $^{1}$H-$^{15}$N SOFAST HMQC spectra of $^{15}$N-labelled IF1-NT at final protein concentration of 30 μM in the absence (red spectra) and presence (black spectra) of 450 μM unlabelled OSCP-CT.
