# Supplementary material for: The mitochondrial inhibitor IF1 binds to the ATP synthase OSCP subunit and protects cancer cells from apoptosis
Source: Cell Death Dis. 2023 Jan 23;14(1):54. doi: 10.1038/s41419-023-05572-y (PMC9870916; doi:10.1038/s41419-023-05572-y)
Supplement: Supplementary file 4 — Figure S4 [file 41419_2023_5572_MOESM4_ESM.pdf]

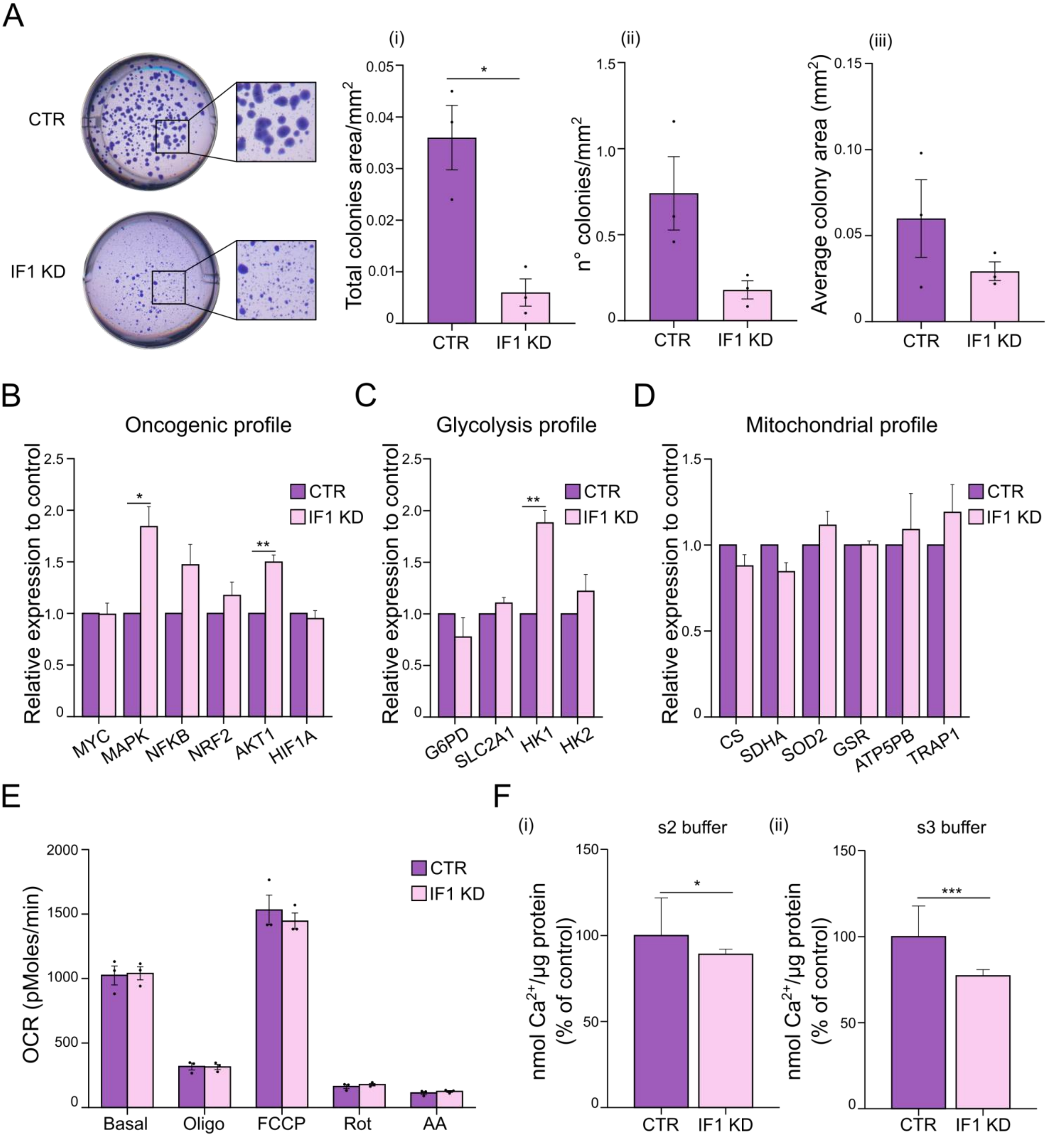
A
CTR
IF1 KD
(i)
Total colonies area/mm²
CTR
IF1 KD
(ii)
n° colonies/mm²
CTR
IF1 KD
(iii)
Average colony area (mm²)
CTR
IF1 KD
B
Oncogenic profile
Relative expression to control
CTR
IF1 KD
MYC
MAPK
NFKB
NRF2
AKT1
HIF1A
C
Glycolysis profile
Relative expression to control
CTR
IF1 KD
G6PD
SLC2A1
HK1
HK2
D
Mitochondrial profile
Relative expression to control
CTR
IF1 KD
CS
SDHA
SOD2
GSR
ATP5PB
TRAP1
E
OCR (pMoles/min)
CTR
IF1 KD
Basal
Oligo
FCCP
Rot
AA
F
(i)
s2 buffer
nmol Ca2+/μg protein (% of control)
CTR
IF1 KD
(ii)
s3 buffer
nmol Ca2+/μg protein (% of control)
CTR
IF1 KD

Figure S4

A. Soft agar assay is shown of CTR and IF1 knock down (KD) HeLa cells. Cells are grown for 15 days and then stained for the quantification analysis. Left panels, representative images of CTR and IF1 KD cells in stained wells and their magnification. Right panels, mean of the (i) total colony area/mm^2, (ii) number of colonies/mm^2 and (iii) average of colony area in mm^2. Data are mean ± SEM of 3 independent experiments, *p = 0.011.

B-C-D. Analysis of mRNA relative expression in CTR and IF1 KD HeLa cells. The mRNA levels are analyzed for a selected number of genes involved in (B) oncogenic, (C) glycolytic, and in (D) mitochondrial profiles that are shown. Values are normalized to their levels in CTR and are mean of 3 independent experiment ± SEM, *p = 0.035, **p < 0.01.

E. Oxygen consumption rate (OCR) is shown of CTR and IF1 KD HeLa cells. OCR is measured before (basal) and after treatment with oligomycin (oligo), carbonyl cyanide p-(trifluoromethoxy) phenylhydrazone (FCCP), rotenone (Rot) and antimycin A (AA). Mean OCR measurement ± SEM is shown of adherent HeLa cells *in situ*, seeded at the concentration of 50000 cells/well. Data are from 4 independent experiments.

F. Calcium retention capacity (CRC) is assessed in CTR and IF1 KD permeabilized HeLa cells in a buffer promoting state 2 (i) or state 3 (ii) respiration and containing the membrane impermeable Ca^{2+} sensor, Ca^{2+} Green-5N. Histograms represent nmols of Ca^{2+} per µg of protein that are necessary to cause PTP opening. Data are mean (expressed as % of controls) ± SEM of 4 or 8 experiments in (i) or (ii), respectively. *P values* are *p= 0.05, ***p= 0.0004.
