# Supplementary material for: The mitochondrial inhibitor IF1 binds to the ATP synthase OSCP subunit and protects cancer cells from apoptosis
Source: Cell Death Dis. 2023 Jan 23;14(1):54. doi: 10.1038/s41419-023-05572-y (PMC9870916; doi:10.1038/s41419-023-05572-y)
Supplement: Supplementary file 5 — Figure S5 [file 41419_2023_5572_MOESM5_ESM.pdf]

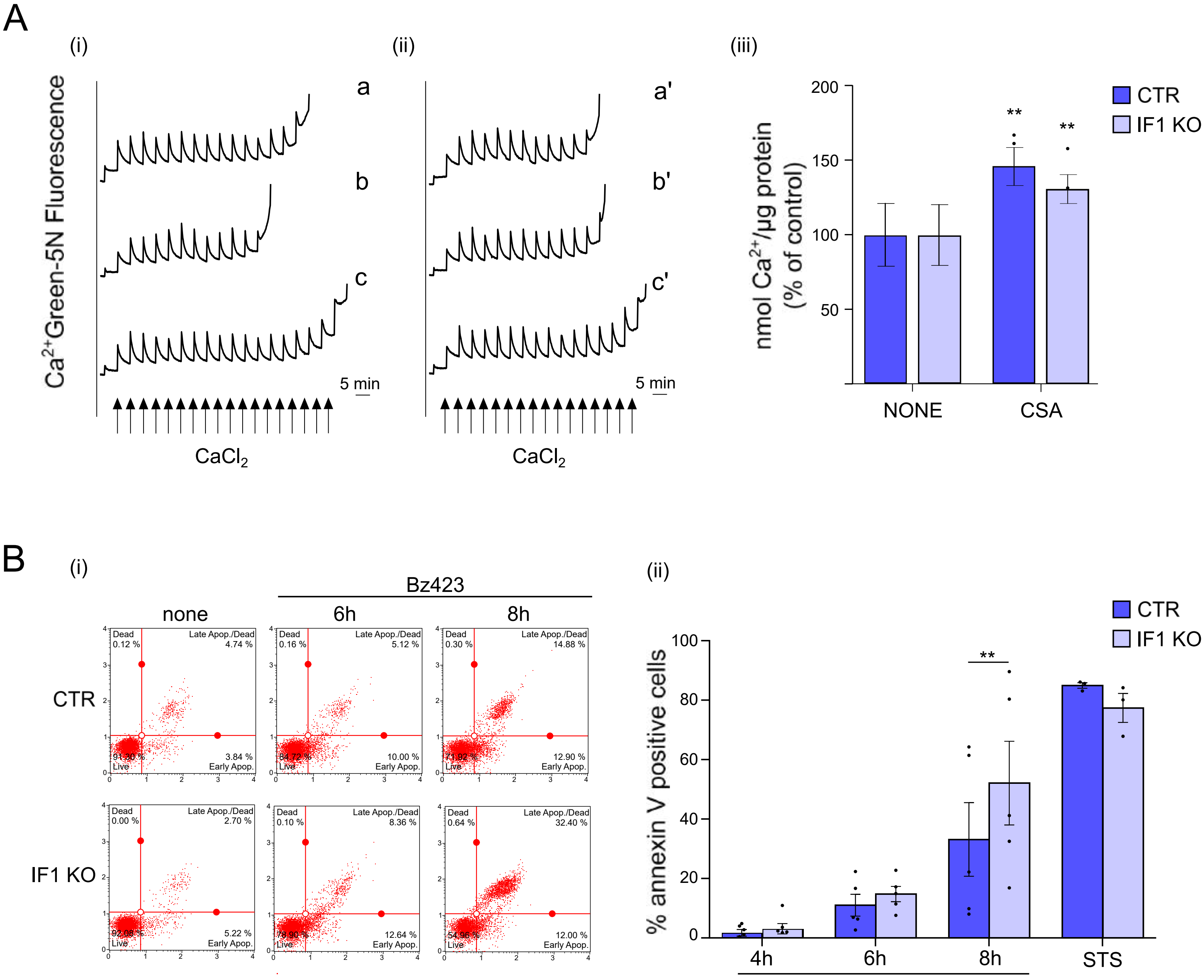

A
(i)
(ii)
(iii)
Ca2+Green-5N Fluorescence
a
b
c
a'
b'
c'
5 min
5 min
CaCl2
CaCl2
nmol Ca2+/µg protein
(% of control)
200
150
100
50
**
**
CTR
IF1 KO
NONE
CSA
B
(i)
(ii)
Bz423
none
6h
8h
CTR
IF1 KO
Dead
Late Apop./Dead
Live
Early Apop.
0.12 %
4.74 %
3.84 %
0.16 %
5.12 %
10.00 %
0.30 %
14.88 %
12.90 %
0.00 %
2.70 %
5.22 %
0.10 %
8.36 %
12.64 %
0.64 %
32.40 %
12.00 %
% annexin V positive cells
100
80
60
40
20
0
**
CTR
IF1 KO
4h
6h
8h
STS
Bz423

Figure S5

A. Ca^{2+} retention capacity (CRC) is assessed in permeabilized CTR (i) and IF1 KO (ii) HeLa cells in a synthesis-based medium containing respiratory substrates and the membrane-impermeable Ca^{2+} sensor Ca^{2+} Green-5N in the presence of 1 mM phosphate (a; a'); 5 mM phosphate (b; b') or 1.6 μM CsA and 5 mM phosphate (c; c'). Ca^{2+} Green-5N fluorescence was monitored following repeated additions of 5 μM Ca^{2+} pulses (bottom arrows). A return of Ca^{2+} Green-5N fluorescence to baseline reflects uptake of Ca^{2+} by mitochondria, whereas a sudden increase in fluorescence is indicative of PTP opening. One experiment representative of 4 for CTR (i) and IF1 KO (ii) is shown. Ca^{2+} Green-5N fluorescence baselines are graphically shifted upward to avoid trace overlap.

In (iii), the histogram represents nmols of Ca^{2+} per μg of protein retained by CTR (dark bars) and IF1 KO (light bars) cells, that are kept as in b, b' (NONE) or in the presence of 1.6 μM CsA as in c, c' (CSA). Data represent the mean (expressed as % of controls) ± SEM (4 independent experiments run in triplicate each condition), *P value* is **p=0.011 for CTR and 0.019 for IF1 KO.

B. Adherent CTR and IF1 KO HeLa cells are treated with 100 μM Bz423 for 4, 6, 8 h, or 2 μM Staurosporine (STS) for 24 h, collected and incubated with annexin V-fluorescent probe to quantify apoptotic cells by cytofluorimetric measurements. Representative cytofluorimetric dot-plot profiles (i) of CTR and IF1 KO cells are shown after 6 h or 8 h of Bz423 treatment, or in the absence of treatment (none). In (ii), histograms show the mean quantification of apoptotic annexin V-positive CTR and IF1 KO cells (expressed as % of the total cell population including living and dead cells). Data are from 5 or 3 independent experiments ±SEM for Bz423 or STS treatments, respectively. *P value* is **p = 0.0043.
