# Supplementary material for: The mitochondrial inhibitor IF1 binds to the ATP synthase OSCP subunit and protects cancer cells from apoptosis
Source: Cell Death Dis. 2023 Jan 23;14(1):54. doi: 10.1038/s41419-023-05572-y (PMC9870916; doi:10.1038/s41419-023-05572-y)
Supplement: Supplementary file 6 — Table S1 [file 41419_2023_5572_MOESM6_ESM.docx]

| Pathways | Gene name | Primer Sequence (5′-3′) | Amplicon Length (bp) |
| --- | --- | --- | --- |
| Glicolysis | ***G6PD*** | F: GAAGAGCTTTTCCAGGGCG  R: GATGAAGGTGTTTTCGGGCA | **150** |
|  | ***SLC2A1*** | F:CACTGGAGTCATCAATGCCC  R:GAGAAGGAGCCAATCATGCC | **159** |
|  | ***HK1*** | F:GGATCCCTCAACCCTGGAAA  R:TTCTTTGGCATTGTGGAGGC | **213** |
|  | ***HK2*** | F:GACCAACTTCCGTGTGCTTT  R:TCCATGAAGTTAGCCAGGCA | **156** |
| Mitochondrial profile | ***CS*** | F:ATGGCTTTACTTACTGCGGC  R:AGTCTTAATTCTGGCCTGCTC | **150** |
|  | ***SDHA*** | F:GAACATCGGAACTGCGACTC  R:CCCACAACCTTCTTGCAACA | **92** |
|  | ***SOD2*** | F:CGTGAACAACCTGAACGTCA  R:CTTCCAGCAACTCCCCTTTG | **185** |
|  | ***GSR*** | F:ACACAGCTGTCCACTCTGAA  R:CATGGCCACGGATGATTTCT | **177** |
|  | ***ATP5F1B*** | F:CCCTGTACCACCTCTTCCTG  R:AGGTCTCTGCGCTAATCACA | **173** |
|  | ***TRAP1*** | F:CCAAGCACTGCCAGAAATGG  R:GGACACCAGCTCTTCCTGTG | **106** |
| Oncogenic profile | ***MYC*** | F:TTCGGGTAGTGGAAAACCAG  R:CAGCAGCTCGAATTTCTTCC | **203** |
|  | ***MAPK1*** | F:CGCTACACCAACCTCTCGTA  R:TAGGTCTGGTGCTCAAAGGG | **122** |
|  | ***NFKB1*** | F:GGACTACCTGGTGCCTCTAG  R:GCAGATCCCATCCTCACAGT | **168** |
|  | ***NFE2L2*** | F:ATGATGGACTTGGAGCTGC  R:GCTCATACTCTTTCCGTCGC | **142** |
|  | ***AKT1*** | F:ATTGTGAAGGAGGGTTGGCT  R:TTGTAGCCAATGAAGGTGCC | **101** |
|  | ***HIF1A*** | F:TGCTTACACACAGAAATGGCC  R:ATACGTGAATGTGGCCTGTG | **162** |
| Reference Gene | ***GUSB*** | F:GAAAATACGTGGTTGGAGAGCTCATT  R:CCGAGTGAAGATCCCCTTTTTA | **101** |

**Supplementary Table 1.** List of genes and primer sequences used for RT-PCR analysis in HeLa IF1 KO, IF1 KD and control cells.
