# Supplementary material for: The mitochondrial inhibitor IF1 binds to the ATP synthase OSCP subunit and protects cancer cells from apoptosis
Source: Cell Death Dis. 2023 Jan 23;14(1):54. doi: 10.1038/s41419-023-05572-y (PMC9870916; doi:10.1038/s41419-023-05572-y)
Supplement: Supplementary file 7 — Original blot [file 41419_2023_5572_MOESM7_ESM.pdf]

FIGURE 1A

(i)

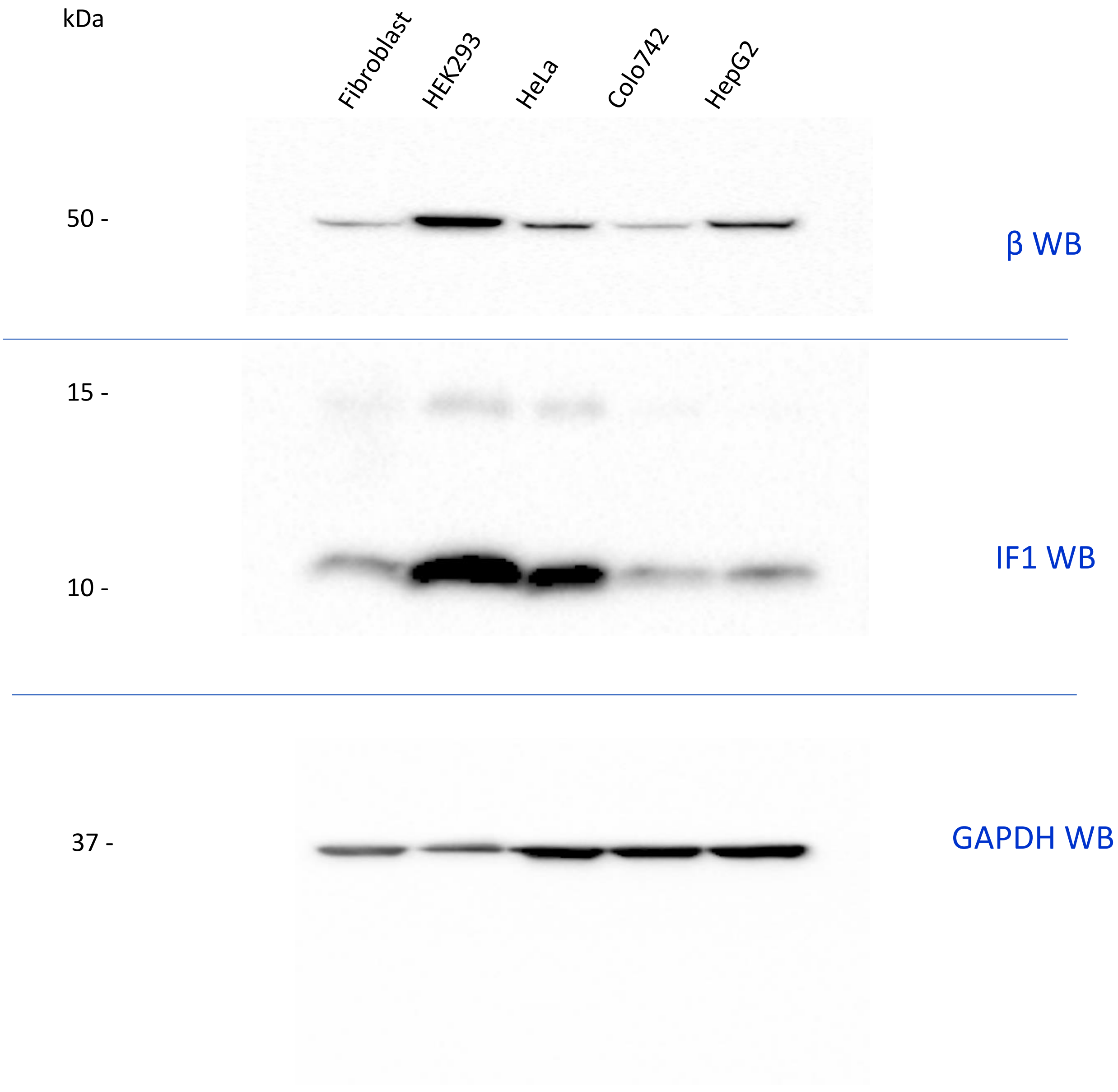
kDa
Fibroblast
HEK293
HeLa
Colo742
HepG2
50 -
β WB
15 -
IF1 WB
10 -
37 -
GAPDH WB

FIGURE 1B

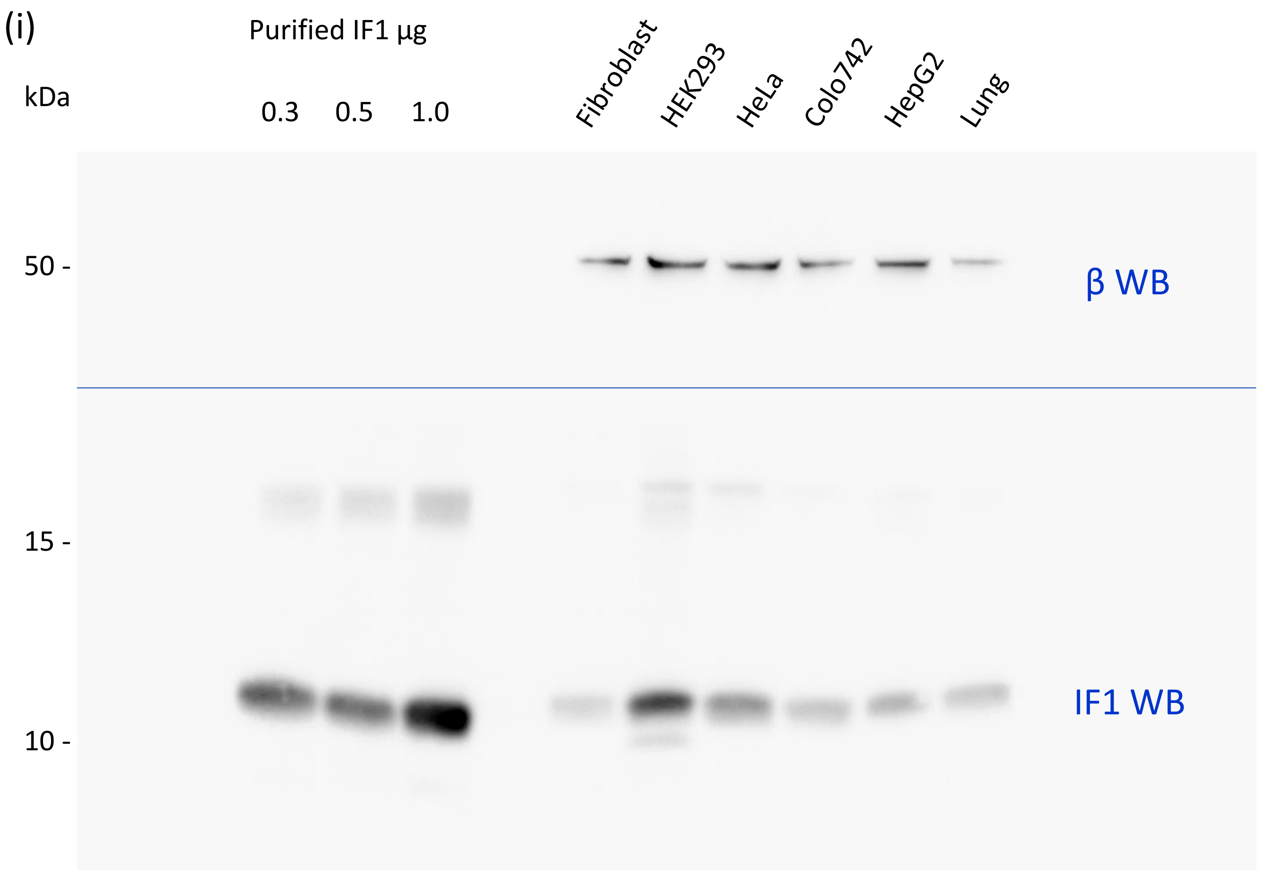
(i)
Purified IF1 µg
kDa
0.3
0.5
1.0
Fibroblast
HEK293
HeLa
Colo742
HepG2
Lung
50 -
β WB
15 -
10 -
IF1 WB

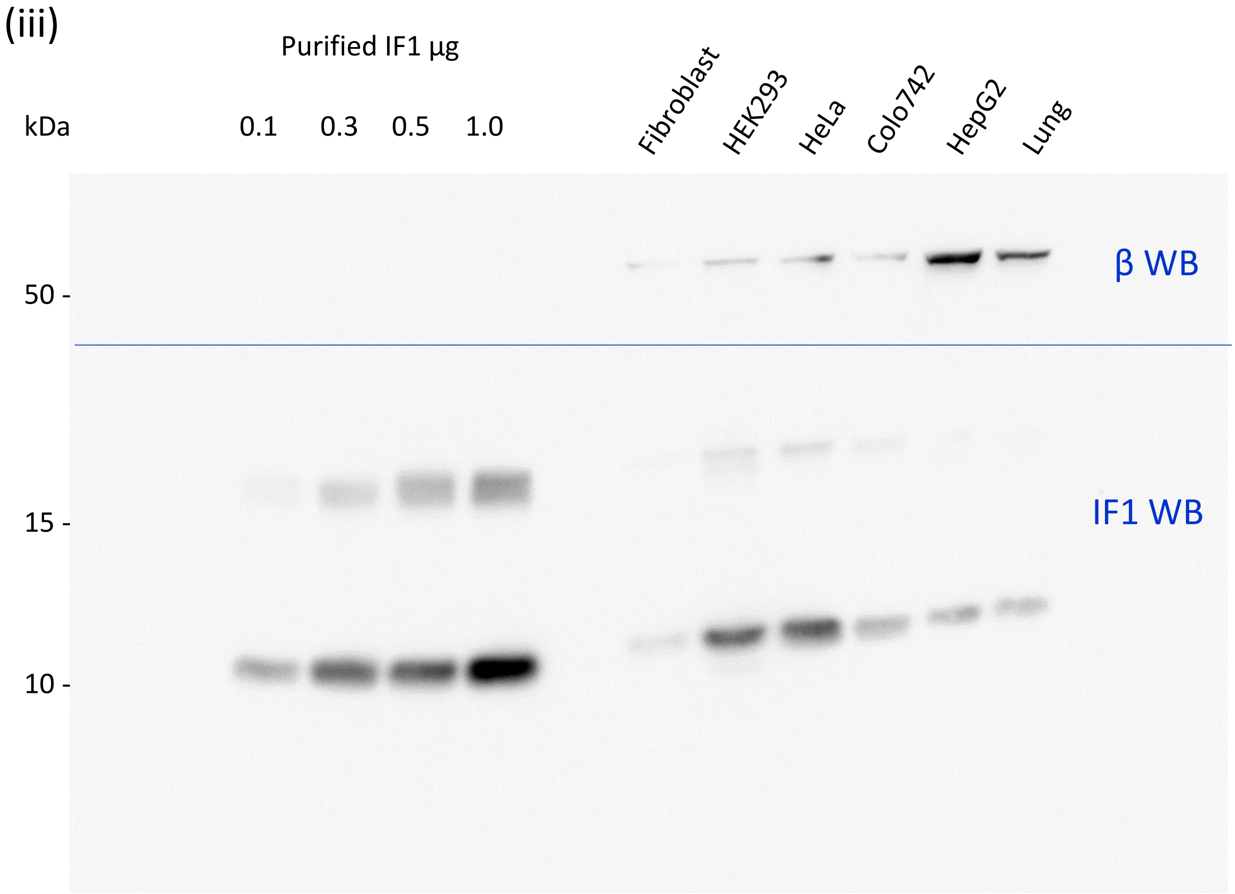
(iii)
Purified IF1 µg
kDa
0.1
0.3
0.5
1.0
Fibroblast
HEK293
HeLa
Colo742
HepG2
Lung
50 -
β WB
15 -
IF1 WB
10 -

FIGURE 1D

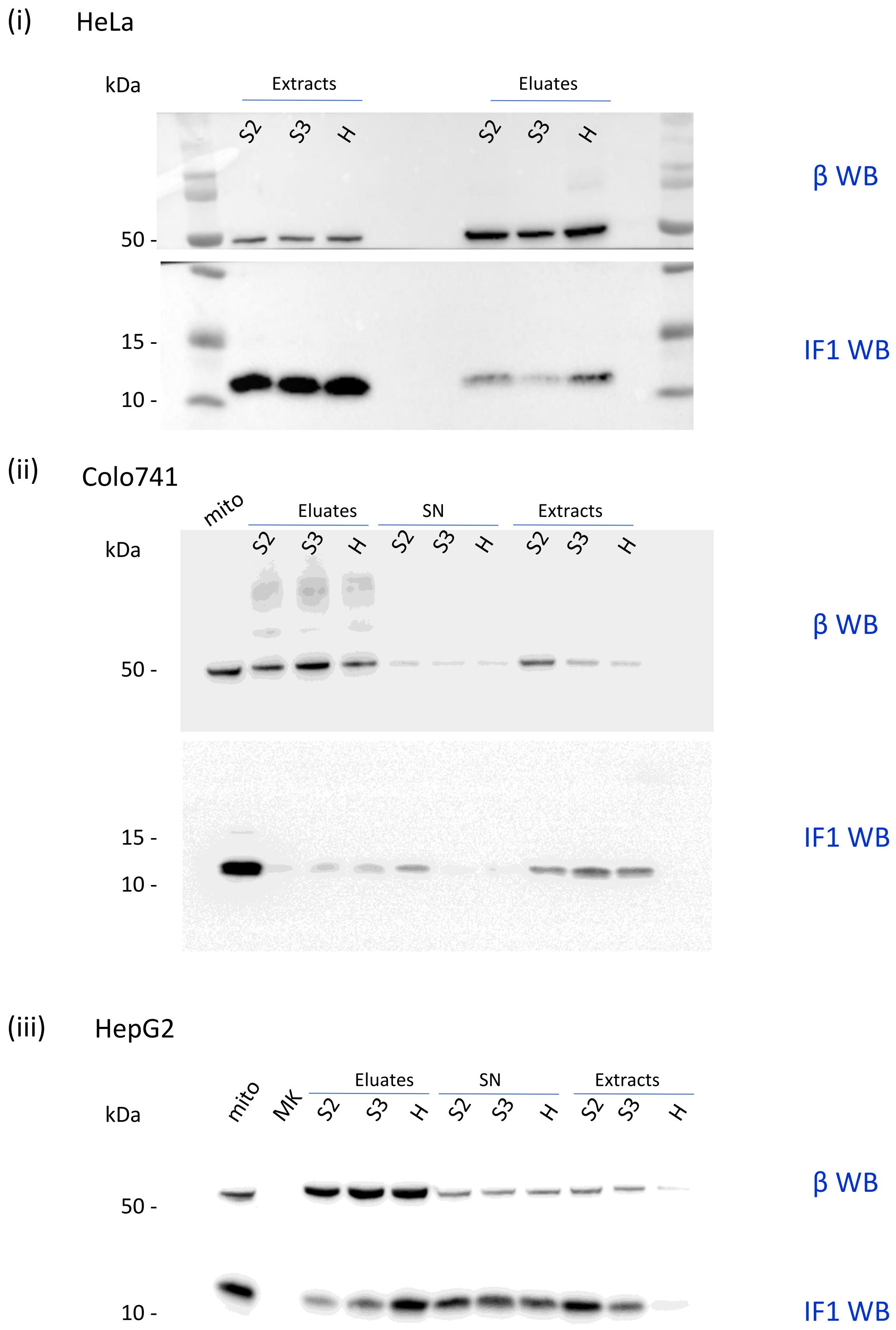

(i)
HeLa
kDa
Extracts
Eluates
S2
S3
H
S2
S3
H
β WB
50 -
15 -
10 -
IF1 WB
(ii)
Colo741
mito
Eluates
SN
Extracts
kDa
S2
S3
H
S2
S3
H
S2
S3
H
β WB
50 -
15 -
10 -
IF1 WB
(iii)
HepG2
mito
MK
Eluates
SN
Extracts
kDa
S2
S3
H
S2
S3
H
S2
S3
H
β WB
50 -
10 -
IF1 WB

FIGURE 2B

(i)

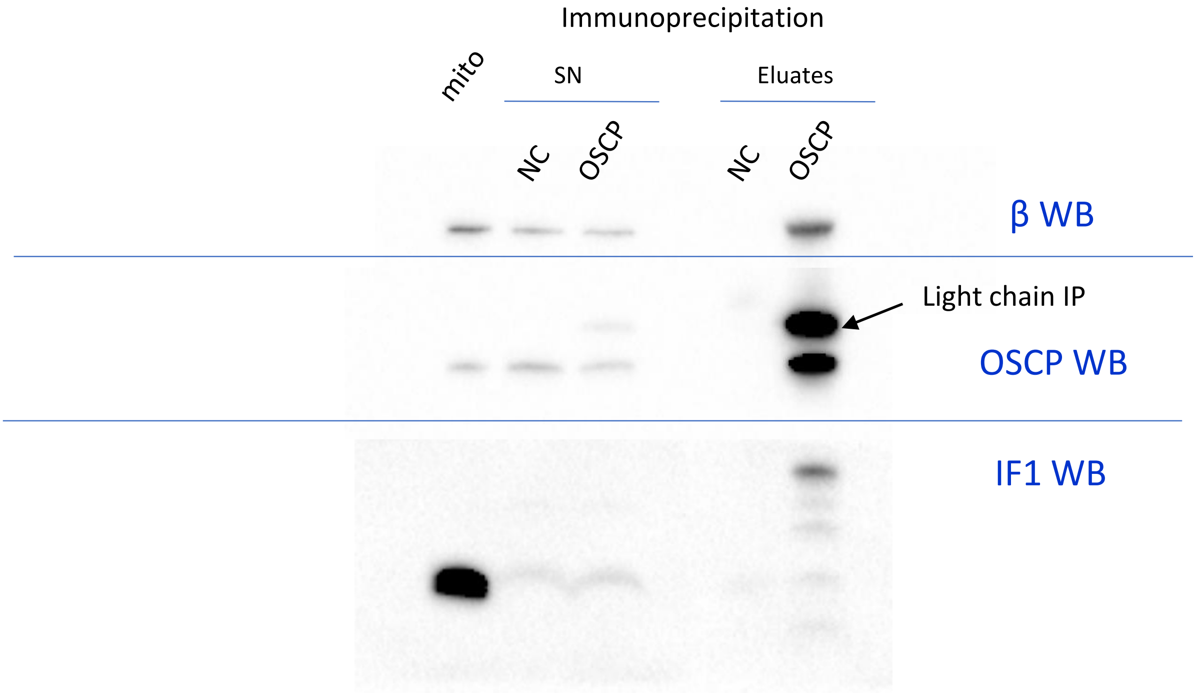

(ii)

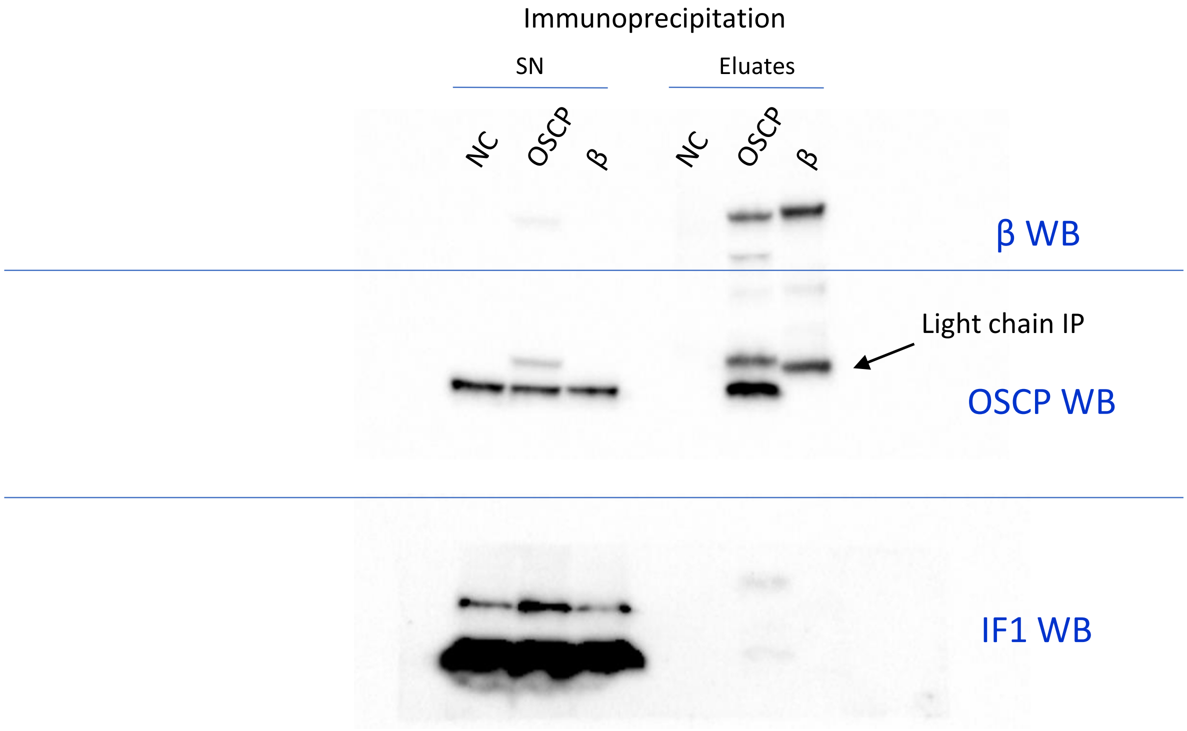

FIGURE 6B

(i)

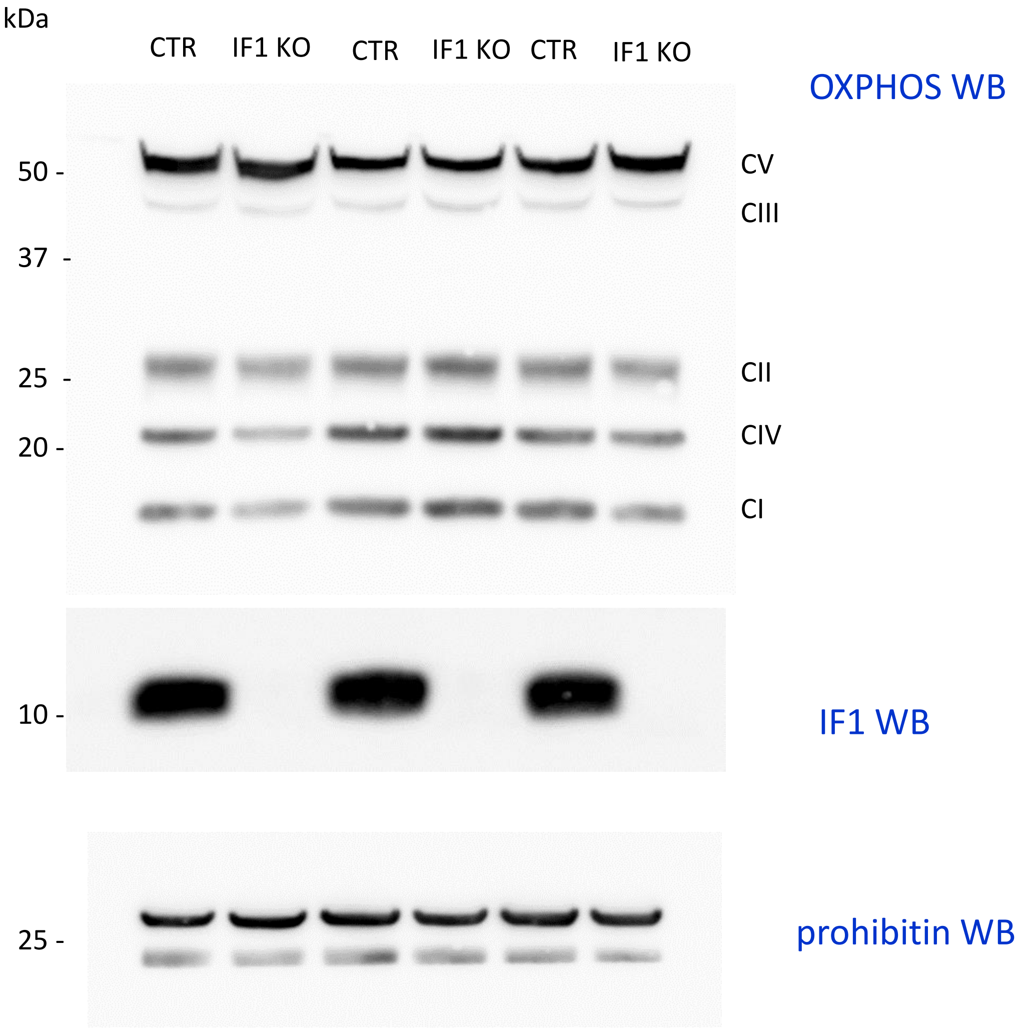
kDa
CTR
IF1 KO
CTR
IF1 KO
CTR
IF1 KO
OXPHOS WB
50 -
37 -
25 -
20 -
CV
CIII
CII
CIV
CI
10 -
IF1 WB
25 -
prohibitin WB

FIGURE 6D

(i)

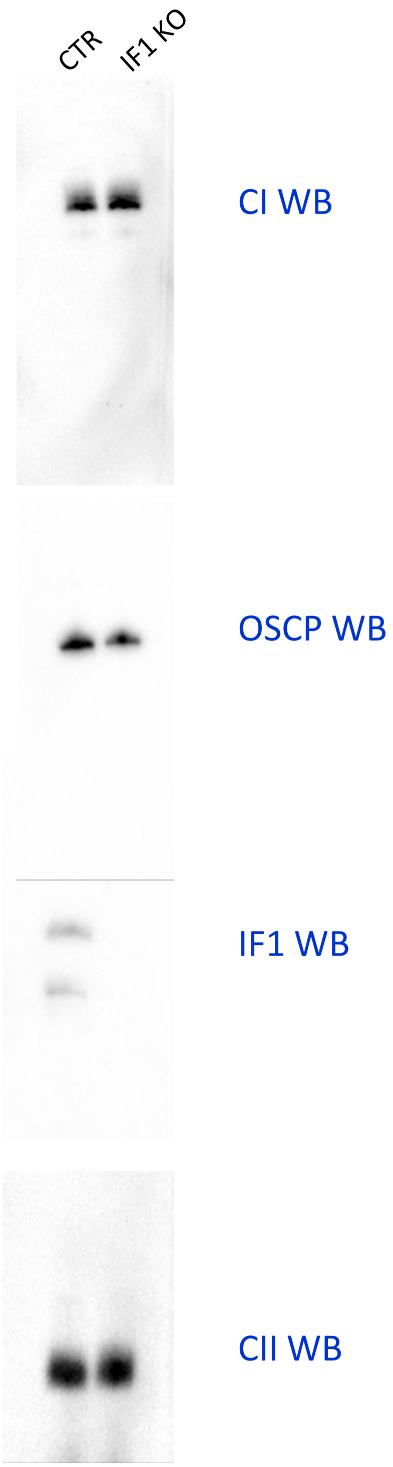

FIGURE 7C

(i)

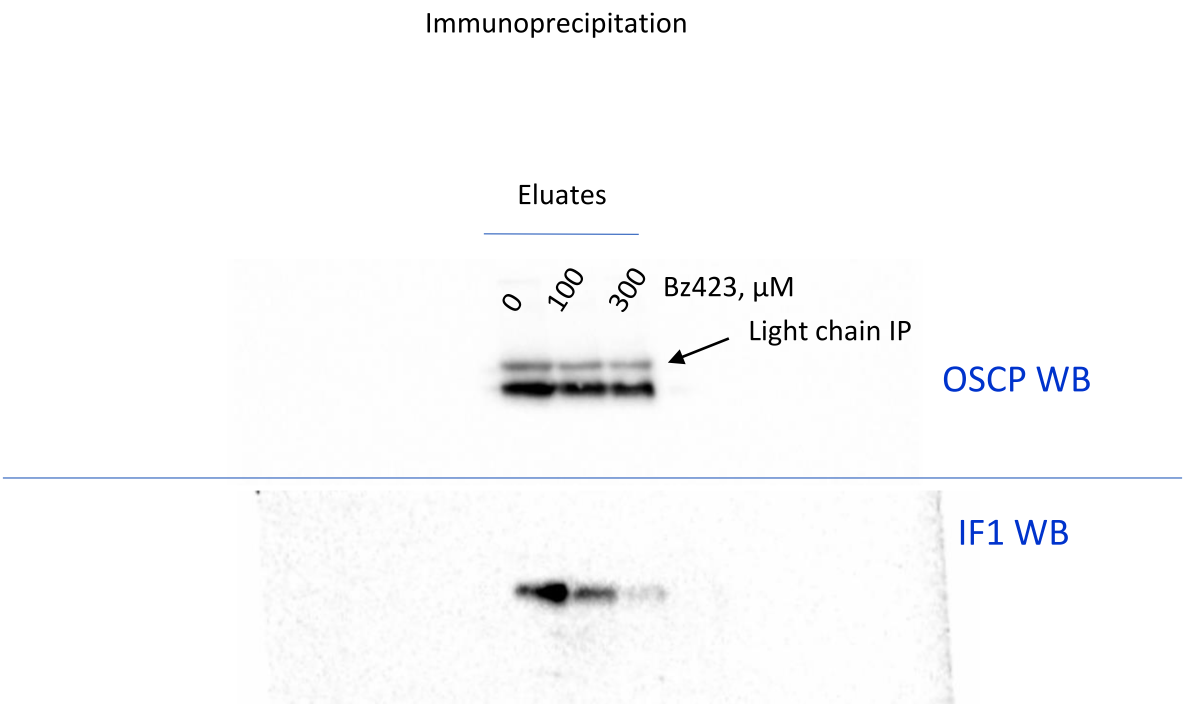
Immunoprecipitation
Eluates
0
100
300
Bz423, µM
Light chain IP
OSCP WB
IF1 WB

FIGURE 8A

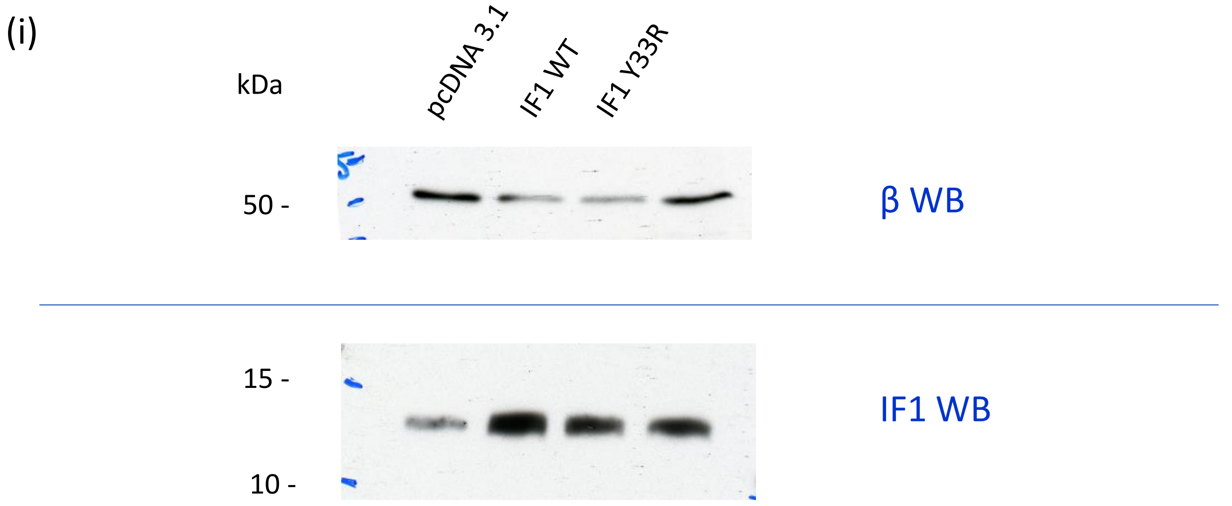

FIGURE 8B

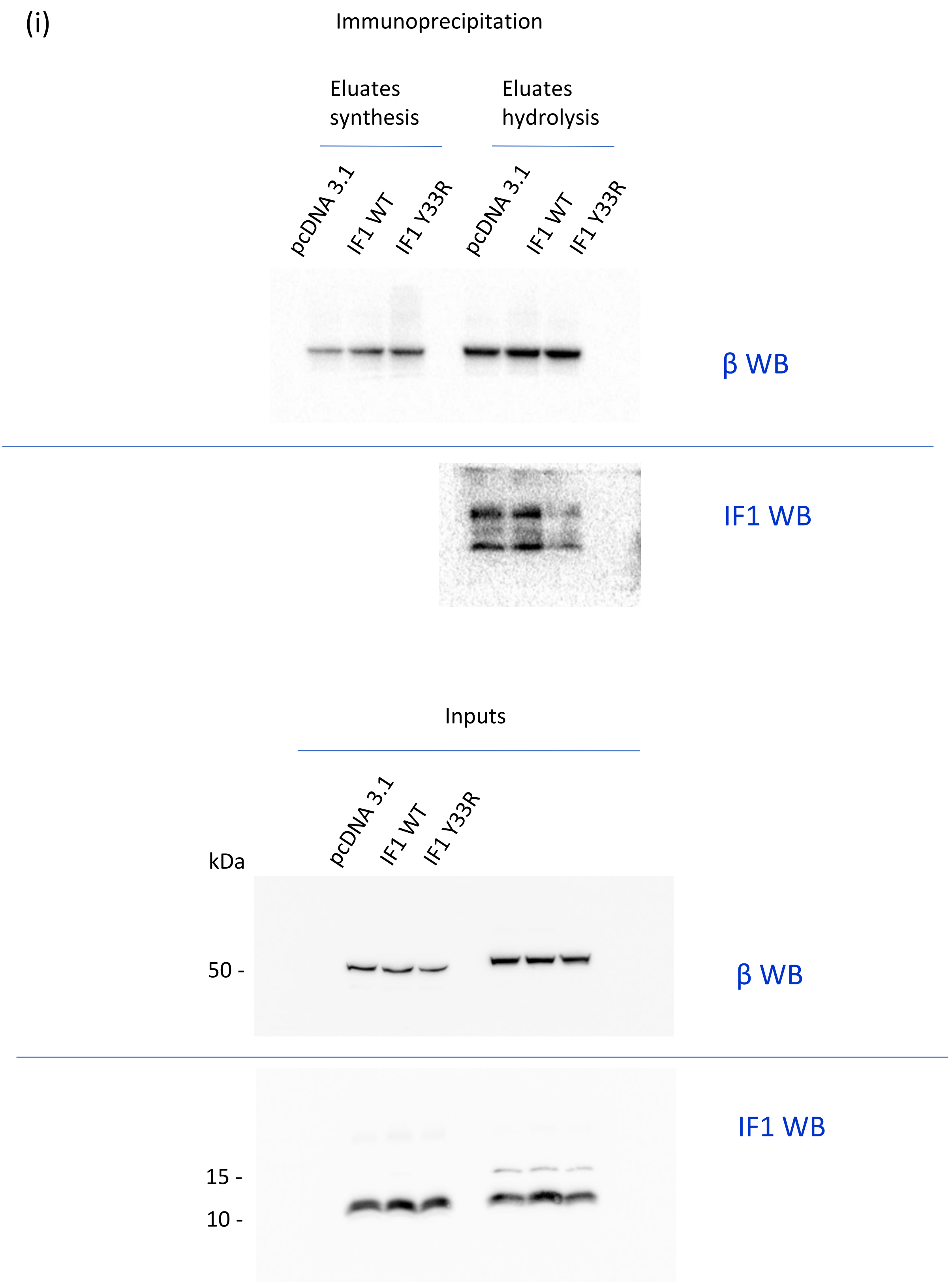

(i)
Immunoprecipitation
Eluates synthesis
Eluates hydrolysis
pcDNA 3.1
IF1 WT
IF1 Y33R
pcDNA 3.1
IF1 WT
IF1 Y33R
β WB
IF1 WB
Inputs
pcDNA 3.1
IF1 WT
IF1 Y33R
kDa
50 -
β WB
15 -
10 -
IF1 WB

FIGURE 8C

(i)

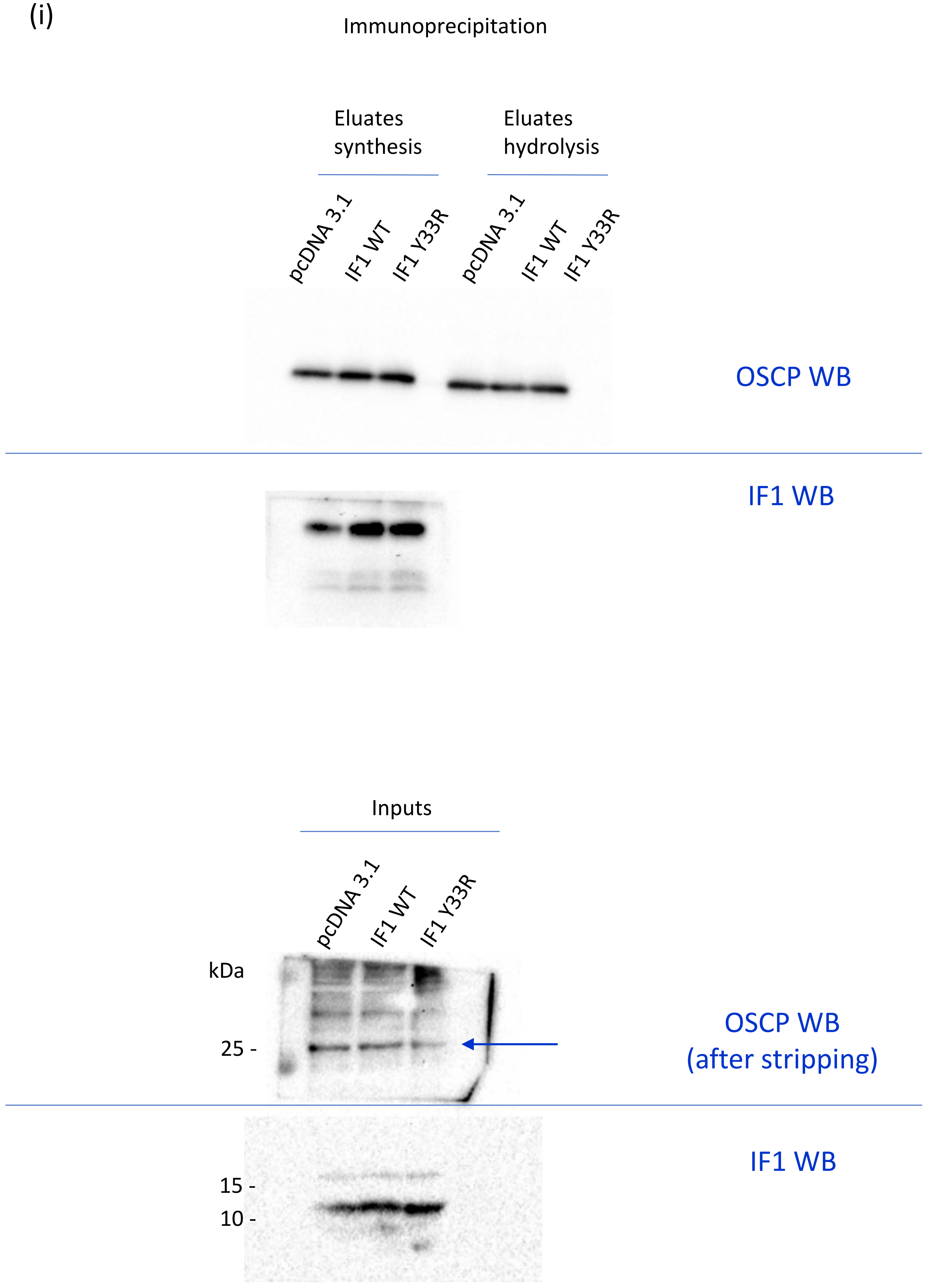

Immunoprecipitation
Eluates synthesis
Eluates hydrolysis
pcDNA 3.1
IF1 WT
IF1 Y33R
pcDNA 3.1
IF1 WT
IF1 Y33R
OSCP WB
IF1 WB
Inputs
pcDNA 3.1
IF1 WT
IF1 Y33R
kDa
25 -
OSCP WB
(after stripping)
15 -
10 -
IF1 WB
